# Supplementary material for: Gut microbiomes of wild great apes fluctuate seasonally in response to diet
Source: Nat Commun. 2018 May 3;9:1786. doi: 10.1038/s41467-018-04204-w (PMC5934369; doi:10.1038/s41467-018-04204-w)
Supplement: Supplementary file 1 — Supplementary Information [file 41467_2018_4204_MOESM1_ESM.pdf]

Supplementary Information

Gut Microbiomes of Wild Great Apes Fluctuate Seasonally in Response to Diet

Hicks et al.

SUPPLEMENTARY NOTES

Supplementary Note 1:

We obtained a total of 3,236,079 raw 16S rRNA gene sequences for the WLG dataset and 803,566 raw sequences for the chimpanzee dataset. After quality filtering, chimera detection, removal of PyNAST alignment failures, and chloroplast sequence exclusion, 2,465,594 and 412,713 bacterial sequences remained in the final quality-filtered dataset and operational taxonomic unit (OTU) table for the 87 WLG and 18 chimpanzee fecal samples, respectively. The OTU tables generated by open-reference OTU clustering (97%) with QIIME revealed a total of 3,320 OTUs in the WLG dataset (**Supplementary Data 2**) and 2,340 OTUs in the chimpanzee dataset (**Supplementary Data 3**). There was an average of 525 OTUs per individual WLG (range = 177-892) and 821 OTUs per individual chimpanzee (range = 546-1,065), suggesting that the number of bacterial species found in each individual WLG and chimpanzee may be similar to the range reported for humans (500-1000 species)[1].

Supplementary Note 2:

Across WLG samples, the distribution of Firmicutes appeared as a clear gradient, ranging from 15.8% to 91.2% abundance in individual WLG fecal samples (**Supplementary Fig. 2a**). Firmicutes, on average, accounted for nearly half of the relative abundance of all phyla (**Supplementary Fig. 2b**), which was widely distributed among many genera (**Supplementary Data 2**).

Within Chloroflexi, on the other hand, the putative genus *SHD-231* accounted for 99.96% of all sequences (**Supplementary Fig. 2b**). While Chloroflexi was the third most abundant phylum in WLGs, the second most abundant genus (*SHD-231*) (**Fig. 1c**), and present in 100% of WLG samples in this study, it was not found at appreciable levels in any of the other host groups (**Supplementary Data 4**).

Spirochaetes was the fourth most abundant phylum in WLGs (**Fig. 1c**) and present in 100% of WLG samples in this study (**Supplementary Data 4**). The most abundant Spirochaetes genera present in the WLGs were *Treponema* and *Sphaerochaeta* (accounting for 68.16% and 31.35% of all Spirochaetes sequences, respectively) (**Supplementary Fig. 2b**). The mean relative abundances of *Treponema* (7.59%) and *Sphaerochaeta* (3.49%) in WLGs were remarkably high compared to westernized human populations, and their relative abundance ranged widely among individual samples (0.004-57.32% for *Treponema* and 0.05-23.95% for *Sphaerochaeta*) (**Supplementary Data 4**). Similar to WLGs, Spirochaetes prevalence and relative abundance were also high in sympatric chimpanzees, and the relative abundances of *Treponema*

(0.06-24.23%) and *Sphaerochaeta* (0.09-15.54%) in individual samples ranged widely. However, in contrast to WLGs, the mean relative abundance of *Sphaerochaeta* (4.86%) was higher than for *Treponema* (1.92%) (**Supplementary Data 4**). Compared to the African great apes, Spirochaetes were similarly prevalent, but present at lower relative abundance in Old World monkeys (**Supplementary Fig. 1b-e** and **Supplementary Data 4**). In contrast to NHPs, humans from the U.S. and urban Mongolia had extremely low prevalence of Spirochaetes (1.7% and 3.08% prevalence, respectively), and in those few individuals, relative abundance did not exceed 0.02% in U.S. humans and 2.87% in urban Mongolians. *Sphaerochaeta*, the most abundant Spirochaete in our closest living relative, the chimpanzee, was completely absent from the humans examined in this study.

Supplementary Note 3:

Partitioning around medoids (PAM) clustering of all primate groups, based on the square root Jensen-Shannon divergence (rJSD), as well as BC dissimilarity, among genus-level relative abundance distributions, as described by Arumugam *et al*.[2], most strongly supported three clusters based on the Calinski-Harabasz (CH) index and the average Silhouette (SI) width score (**Supplementary Fig. 4b** and **5a-b**). Nearly all of the human samples were in clusters 1-2, which were defined by the known human enterotype-associated taxa, *Prevotella* and *Bacteroides*, respectively (**Supplementary Figs. 4b** and **5c**). In contrast, nearly all WLGs, chimpanzees, and Old World monkeys formed a single cluster, cluster 3, defined by *Bifidobacterium* and the two Spirochaete genera, *Treponema* and *Sphaerochaeta*. These results suggest that sympatric African great apes and allopatric Old World monkeys have microbiota composition more similar to each other than to either human population, ultimately indicating that diet may be a greater determinant of microbiome composition than host phylogenetic relationships. The overwhelming majority (92%) of U.S. humans fell into the *Bacteroides*-defined cluster 2, while Mongolian humans were more prevalent in the *Prevotella*-defined cluster 1 (88%) (**Supplementary Fig. 4b**). The third cluster, defined by *Bifidobacterium, Treponema,* and *Sphaerochaeta* and containing the vast majority of NHP samples, separated from both human populations along PC2, but was closer to the Mongolian human-dominated *Prevotella* cluster along PC1. Similarly, principal coordinate analysis based on the BC dissimilarity among genus-level relative abundance demonstrated clustering of NHPs away from humans along PC2, while the majority of U.S. humans separated remarkably from NHPs and the majority of Mongolian humans along PC1 (**Fig. 1e**). As the *Prevotella* enterotype has been linked to plant-based, carbohydrate-rich diets[3], it is perhaps

surprising that the vast majority of Mongolian humans, whose diet is characterized by high consumption of animal products[4], were assigned to the *Prevotella* enterotype. However, food frequency questionnaire results indicate that in addition to meat and fermented dairy products, this study population also consumes large quantities of fruits, vegetables, ginseng, and other plant-based food items, which, collectively, account on average for over 50% of total diet[4].

To further contrast the microbiota between great apes, Old World monkeys, and human populations, we evaluated inter-group BC dissimilarity based on the genus-level relative abundance of the microbiota (**Supplementary Fig. 4c**). The microbiotas of all three NHP species (WLGs, chimpanzees, and baboons) were more dissimilar to U.S. humans than Mongolian humans. Despite the closer phylogenetic relationship between WLGs and humans compared to baboons and humans, the WLG microbiota was more dissimilar to both human populations. This was further supported by PCoA based on the unweighted UniFrac metric, which showed greater separation of WLGs from humans along PC1 compared to baboons and other Old World Monkeys (**Supplementary Fig. 4d**). Sympatric WLGs and chimpanzees had the lowest dissimilarity. The dissimilarity between these two different sympatric animal species was even lower than the dissimilarity between two human populations (**Supplementary Fig. 4c**). While this further supports other findings suggesting that microbial communities in sympatric great apes converge[5], it additionally indicates that shared environment, and likely diet, drive greater convergence of the microbiota of two phylogenetically distinct African great apes, than is observed between two populations of the same species (*H. sapiens*) with highly varied diets.

Supplementary Note 4:

In our analyses using PAM clustering based on the rJSD among genus-level relative abundance distributions, both the CH index and the average SI score most strongly supported four WLG enterotypes and two chimpanzee enterotypes. For the U.S. and Mongolian humans, the CH index most strongly supported three clusters, while the SI score supported two clusters. It has been shown that clustering based on different distance or dissimilarity metrics and taxonomic levels can affect enterotyping, and it has been recommended to use multiple distance metrics to verify the presence of enterotypes[6]. Accordingly, we also performed PAM clustering on each dataset using the BC dissimilarity among genus-level relative abundance distributions (**Supplementary Fig. 5e, 5g, 5i, 5k, 5m**), and, in the case of any discrepancies between enterotypes identified based on rJSD and BC metrics, we evaluated the validity of the clusters based on rJSD and BC with weighted UniFrac distance among species-level (97% OTU approximating species-level) relative abundance distributions. The only

discrepancy between enterotypes identified based on rJSD and BC metrics was for the WLG dataset, with five or nine clusters identified based on the BC metric by CH index and SI score, respectively, compared to the four clusters identified based on the rJSD metric (**Supplementary Fig. 5d-e**). For all other datasets, the optimal number of clusters was consistent between the two distance metrics and the two measures of cluster scores, with the exception of the two human datasets, where the CH index supported three clusters and the SI score supported two clusters for both distance metrics (U.S. human) or for only the rJSD metric (Mongolian human) (**Supplementary Fig. 5j-m)**. However, this is consistent with the two enterotypes, defined by *Bacteroides* and *Prevotella* relative abundance, with an occasional third, defined by the relative abundance of Clostridial genera, that have been previously reported for humans[3,7,8]. As the *Bacteroides* and *Prevotella* enterotypes are most consistently reported for humans and because SI score is an absolute measure of clustering quality, while CH index is only a relative measure[6], we show only the *Bacteroides* and *Prevotella* enterotypes supported by the SI score for the U.S. and Mongolian human datasets.

In order to assess the validity of the different clusterings of WLG samples based on rJSD and BC, we further investigated the enterotype clusters identified with each metric by the weighted UniFrac distance metric. Principal coordinate analysis based on weighted UniFrac distance of species-level (97% OTUs) relative abundance distributions for WLGs was performed, and the distances among samples assigned to each enterotype based on rJSD and BC were assessed (**Supplementary Fig. 7b-d**). Within-cluster UniFrac distances for each enterotype cluster were compared against distances between each other enterotype cluster to assess the validity of the clusters based on rJSD and BC (**Supplementary Fig. 7b-d**). The four WLG clusters based on rJSD were significantly supported (**Supplementary Fig. 7b**). In contrast, neither the five clusters selected based on the CH index from BC nor the nine clusters based on the SI score from BC were validated by consistent significant differences between within-cluster and between-cluster distances (**Supplementary Fig. 7c-d**). Thus, two distance metrics applied to the microbiota of WLG samples at two different taxonomic levels supported four enterotype clusters defined by the relative abundance of *SHD-231*, *Treponema*, *Prevotella*, and *Solibacillus/Staphylococcus*.

It should be noted that based on PAM clustering, at best, only weak support was provided based on SI scores for enterotype clusters obtained for any of the NHP and human groups evaluated in this study, but these scores are consistent in range with previous evaluations of NHP and human enterotypes[2,8,9]. Thus, these enterotypes should be viewed as the optimal clustering based on

Arumugam *et al.*, rather than fitting the definition of enterotypes as statistically supported clusters. Indeed, recent perspective suggests that even in the absence of strong statistical support, enterotyping can provide useful insights[10].

The WLG enterotypes were widely distributed across the geographical sampling area (**Supplementary Fig. 8a**), and there was no significant association between pairwise BC dissimilarity and distance between locations of sample collection within enterotype groups (**Supplementary Fig. 8b**), suggesting that, within this WLG population, enterotypes are not significantly associated with geographic location.

Supplementary Note 5:

In humans, long-term dietary patterns are associated with enterotypes[3]. Given that WLGs must respond to fluctuations in the availability of food resources throughout the year, we investigated monthly variation in the composition of the WLG microbiota. LEfSe analysis identified bacterial taxa that were significantly associated with each month or month group even after adjusting for potentially confounding effects of collection year, collection site latitude and longitude, and WLG gender. Bacterial taxa with relative abundance associated with month of sample collection were spread across seven phyla and were represented at taxonomic levels ranging from phylum to species (**Fig. 3a-b**). Three of the top six most abundant phyla in WLGs were associated with month of sample collection (**Fig. 1c** and **3a**-**b**). The relative abundance distributions of the top sampling month-associated bacterial taxa (highest LDA score) suggested that many of these, such as Firmicutes, Clostridiales, and unclassified *Mogibacteriaceae*, were not simply represented by monthly spikes in abundance, but rather, their abundance undulated with peaks and troughs across months that ultimately peaked in the months for which they were identified (**Fig. 3c**). Such distributions may be more indicative of a pattern defined by seasonal trends rather than rapid monthly increases and decreases in abundance. Perhaps one exception may be *Treponema*, the top May biomarker that rose dramatically in abundance from March to May and then declined dramatically in June (**Fig. 3c**, left panel). While no other taxa whose relative abundance defines enterotypes were identified as monthly biomarkers, the undulating distribution of most of the sampling month-associated bacterial taxa suggests that temporal variation may follow trends that are not simply demarcated from one month to the next. Thus, we evaluated the monthly distributions of all genera that defined enterotypes to further investigate patterns of temporal variation (**Supplementary Fig. 9a**). *SHD-231* (enterotype 1) was present at high relative abundance across many months, but dropped in abundance in April and May, when *Treponema* (enterotype 2) abundance spiked, and was also at relatively low abundance in

Jan-Feb. *Prevotella* (enterotype 3) abundance was low from March through May, rose gradually from June to July-August, and remained elevated thereafter. As enterotype 4, defined by *Solibacillus* and *Staphylococcus*, only contained four WLG individuals, the presence of a seasonal distribution could not be evaluated. Thus, the relative abundance of three of the four WLG taxa that defined enterotype clusters demonstrated some level of monthly variation.

Supplementary Note 6:

The metagenomes of WLG samples with the Chloroflexi enterotype were enriched largely in functions that may be viewed as bacterial maintenance functions, such as purine and pyrimidine metabolism and cell envelope biosynthesis (**Fig. 7** and **Supplementary Data 6**). There is some debate over the cell envelope architecture (diderm vs. monoderm) of members of the Chloroflexi phylum. Classically, Chloroflexi stain Gram-negative, have unusual membrane lipids, and lack lipopolysaccharide (LPS) biosynthesis genes[11]. However, most characterized genomes to date are derived from environmental isolates, including CL500-11, whose genome is enriched in cell wall and capsule biosynthesis genes, and RBG-9, which does possess a complete pathway for peptidoglycan synthesis, adding support to the multi-layered cell envelope structure seen in additional Chloroflexi isolates[12,13].

Based on the 16S rRNA gene sequence, Chloroflexi found in WLGs falls within the mammalian-associated *Anaerolineae* clade (**Supplementary Fig. 6b**). Genomic information is only available for a few mammalian-associated *Anaerolineae* from the human oral cavity. These human-associated Chloroflexi (Chl1-2) were shown to encode LPS biosynthetic enzymes and genes for the production of galactose and rhamnose type O-antigens[14]. The WLG Chloroflexi-abundant (*SHD-231*-defined) enterotype 1 is enriched in pathways for the biosynthesis of dTDP and UDP nucleotide sugars (i.e., dTDP-L-rhamnose, dTDP-4-acetamido-4,6-dideoxy-D-galactose, and UDP-alpha-D-glucuronate), decaprenyl phosphate and LPS-O-antigen biosynthesis (**Fig. 7** and **Supplementary Data 6**), suggesting enrichment of genes associated with Gram-negative cell wall formation[15]. However, enterotype 1 samples were also enriched in pathways for teichoic acid biosynthesis (**Supplementary Data 6**), a feature unique to the peptidoglycan layers of many Gram-positive bacteria. Isolation, characterization, and whole genome sequencing of primate and other mammalian-associated Chloroflexi will be needed to fully appreciate the true structure of the cell envelope of these taxa. The human-associated Chloroflexi from the oral cavity may also scavenge and utilize N-acetylglucosamine (GlcNAc) from lysed bacterial and epithelial cells in their environment as they encode homologs of GlcNAc-6-phosphate deacetylase (NagA) and glucosamine-6-

phosphate deaminase (NagB)[14]. Based on our metagenomic analyses, we found that the WLG enterotype 1 was enriched in NagA at the gene-level, compared to the other three enterotypes, but was not significantly enriched in NagB (**Supplementary Fig. 12a**). Enterotype 1 was also enriched in glycosulfatase that could provide an advantage to this enterotype for the utilization of sulfated carbohydrate compounds (**Supplementary Fig. 12b**).

In addition to metagenomic analyses indicating enrichment in plant fiber degrading pathways and genes in WLG *Treponema* enterotype 2, the highest sequence identity between the most abundant WLG *Treponema* sequences and a characterized species was found for *T. bryantii* with 86-88% sequence identity. This is intriguing, as *T. bryantii* has been shown to act in symbiosis with *Fibrobacter succinogenes* as part of a fibrolytic consortium to accelerate digestion of plant fiber in ruminants[16], and *Fibrobacter* was the second highest ranked biomarker of the *Treponema* enterotype (**Supplementary Fig. 5n**).

In addition to *Prevotella* enterotype 3 enrichment in fucose degradation pathway genes, alpha- and beta-N-acetylglucosaminidases, alpha-N-acetylgalactosaminidase, beta-galactosidase, and beta-glucuronidase (**Fig. 8f** and **Supplementary Fig. 13a-e**), enterotype 3 was also enriched in additional pathways and genes associated with mucin degradation. At the pathway level, mannose metabolism was also enriched in enterotype 3 samples (**Supplementary Data 6**). Mucin desulfation is thought to be a rate-limiting step in mucin degradation, and some strains of *Prevotella*, such as *Prevotella* strain RS2, encode a mucin desulfating sulfatase[17,18]. *SHD-231*-abundant enterotype 1 samples were enriched in total glycosulfatases (**Supplementary Fig. 12b**). However, in order to be active, sulfatases require a critical post-translational modification of an active-site cysteinyl or seryl residue to C(α)-formylglycine, which is catalyzed by anaerobic sulfatase-maturating enzyme encoded by certain bacteria, including *Prevotella* strain RS2[17,19]. Accordingly, enterotype 3 samples were enriched in anaerobic sulfatase-maturating enzyme genes (**Supplementary Fig. 13f**). While we did not find significant enrichment in genes required for mucin N-acetylneuraminic acid degradation and utilization (i.e. genes of the Nan operon) in enterotype 3 samples, genes encoding sialic acid transporters were enriched in enterotype 3 samples (**Supplementary Fig. 13g**), suggesting an enhanced capacity for bacterial import of sialic acid.

Aromatic compounds abound in nature, and plants are their main producers. Plants produce a wide range of aromatic plant secondary metabolites (PSMs; i.e. tannins, flavonoids, coumarins and other phenolics), as well as the structural aromatic polymer lignin. Both aerobic and anaerobic bacteria are important degraders of PSMs and lignin, as plants and animals are limited in this capacity[20]. PSMs can have a variety of adverse effects on herbivorous mammals,

and many mammals adapt their feeding behavior to limit exposures[21-23]. *Solibacillus*/*Staphylococcus*-abundant enterotype 4 is enriched in bacterial aerobic (i.e. beta-ketoadipate pathway, 3,4-dihydroxybenzoate [protocatechuate] biosynthesis, benzoate degradation via hydroxylation, and anthranilate degradation via hydroxylation) and anaerobic (i.e. benzoate degradation via CoA ligation) pathways of PSM degradation as well as general pathways of PSM aromatic compound degradation (i.e. phenol degradation, p-cresol degradation, 4-hydroxyphenylacetate degradation, vanillyl-alcohol degradation, etc.)[20,24]. In addition, pathways for degradation of environmental pollutants including benzene, toluene, xylene, polychlorinated biphenyl, dichloromethane, and atrazine were enriched in enterotype 4 samples. However, some enzymes that allow bacteria to degrade naturally occurring plant aromatic compounds are indiscriminant and can also mediate degradation of structurally similar man-made pollutants (bioremediation)[24].

Supplementary Note 7:

The majority of classified *Urticaceae* marker sequences were divided between two genera, *Urera* and *Poikilospermum* (**Supplementary Data 9**). The fruit, leaves, bark, and pith of *Urera* spp. are reportedly eaten by Eastern lowland gorillas (*Gorilla beringei graueri*), but are not typically reported as a major dietary constituent of WLGs. It is thus surprising that we have detected high relative abundance of *Urticaceae* plant sequences in the feces from WLGs with *Prevotella*-abundant enterotype 3 in this region. *Annonaceae* and *Urticaceae* were found at low relative abundance in feces from WLGs with enterotype 2 (low frugivory-associated *Treponema* abundant), were more intermediate in relative abundance in enterotype 1, consistent with the potential transitional nature of enterotype 1 (*SHD-231* abundant), and were completely absent from the rare enterotype 4 (*Solibacillus*/*Staphylococcus*-abundant).

It is important to note that plant metagenomic analyses come with some limitations. For many of the plant taxa identified here, WLGs are known to consume different parts of the plant (bark, fruit, flowers, leaves, pith, roots, seeds, shoots, stem, young leaves), and different plant parts that may be consumed seasonally, such as fruit, cannot be distinguished based on DNA sequences alone. *Moraceae* for example, including *Morus* spp., are reported as both a seasonally consumed plant and a fallback food of WLGs at nearly every geographical site across West-Central Africa, where WLGs consume the fruit, flowers, seeds, leaves, bark and stem of *Moraceae* plants[25]. There is also some indication that very little DNA from ripe fruit survives digestion through the gastrointestinal tract (< 1%), while DNA encased in a protein-fiber matrix (such as is found in leaves and other non-fruit plant parts) may be better preserved[26]. If

this is indeed the case, then the distribution of plants identified by sequencing in feces may be biased in favor of fibrous plants compared to ripe, succulent fruits even when such fruits dominate the diet. In fact, for one enterotype 3 sample, too few plant marker genes were identified for adequate analysis. Thus, this sample was excluded (note enterotype 3, n=4 in **Fig. 9b-d**). Additional work in this area will be needed to assess the impact of these potential biases.

Supplementary Discussion:

Distinctions between Human and WLG *Prevotella* species

89% of all human *Prevotella* sequences were grouped into OTUs that were classified to the species-level, with the majority of sequences identified as *P. copri* or *P. stercorea*. In contrast, 92% of *Prevotella* sequences from WLGs remained unclassified at the species level, indicating that most *Prevotella* found in WLGs are different species than those found in humans. Consistency in metabolic potential at the genus-level cannot be presupposed[27]. In addition, the distinction between the human and WLG *Prevotella* enterotype may further be dependent on the relationship between *Prevotella* and other mucolytic taxa in the microbiome (e.g., *Lachnospiraceae,* also associated with frugivory in WLGs and chimpanzees[28]).

Additional Discussion on WLG *Solibacillus-Staphylococcus* enterotype

The small number of WLGs (n=4, 4.6% of the sampled population) harboring the *Solibacillus-Staphylococcus* enterotype may reflect atypical feeding behavior. Our findings based on plant sequences in fecal samples suggest that gorillas with enterotype 4 consumed high relative abundance of *Moraceae* plants. In addition to nutritionally important components, plants produce a wide range of plant secondary metabolites (PSMs), including aromatic compounds, monoterpenes, alkaloids, and phenolics, such as tannins, lignins, and flavonoids. Many of these PSMs aid in plant defense, serving as toxins and antifeedants, but may also have beneficial/medicinal properties. Compared to chimpanzees, WLGs have been shown to have higher prevalence and load of intestinal parasites that may also fluctuate seasonally and promote dietary self-medication behaviors that have been observed in great apes and other herbivorous mammals[29,30]. Indeed, many plants eaten by great apes are used in traditional medicine, including *Moraceae* members that have been shown to have anti-helminthic, anti-malarial, anti-leishmania, anti-bacterial, anti-ulcerogenic, and analgesic effects[29]. WLGs are herbivore generalists, consuming nearly 200 species of plants[25,31]. Such a generalist feeding behavior, where a variety of plants are consumed, is thought to circumvent toxicosis by limiting the dose of ingestion of any single PSM[22,23]. Little is known about aberrant feeding behaviors in great apes, especially relating to quantitative evaluation of individual plant taxa consumed[29]. Based on at least one study of WLGs in Cameroon, *Moraceae sp.* contained the highest levels of total phenolics and condensed tannins of the 26 plant species evaluated[31]. Animals are limited in their ability to metabolize aromatic compounds, while some bacteria are well adapted for their degradation

and may even use them as a growth substrate[20]. Indeed, PSMs can modulate the composition and function of the intestinal microbiome through bacteriostatic, bactericidal, and selective growth promoting mechanisms[32-34]. WLG enterotype 4 was quite different in both bacterial composition and metabolic function compared to the other three enterotypes. Our findings demonstrating enterotype 4 enrichment in predicted bacterial metabolic pathways for aromatic compound and xenobiotic degradation including phenolics-, alkaloids- and lignin-associated pathways combined with the high relative abundance of *Moraceae* plant sequences in their stool add support to this hypothesis.

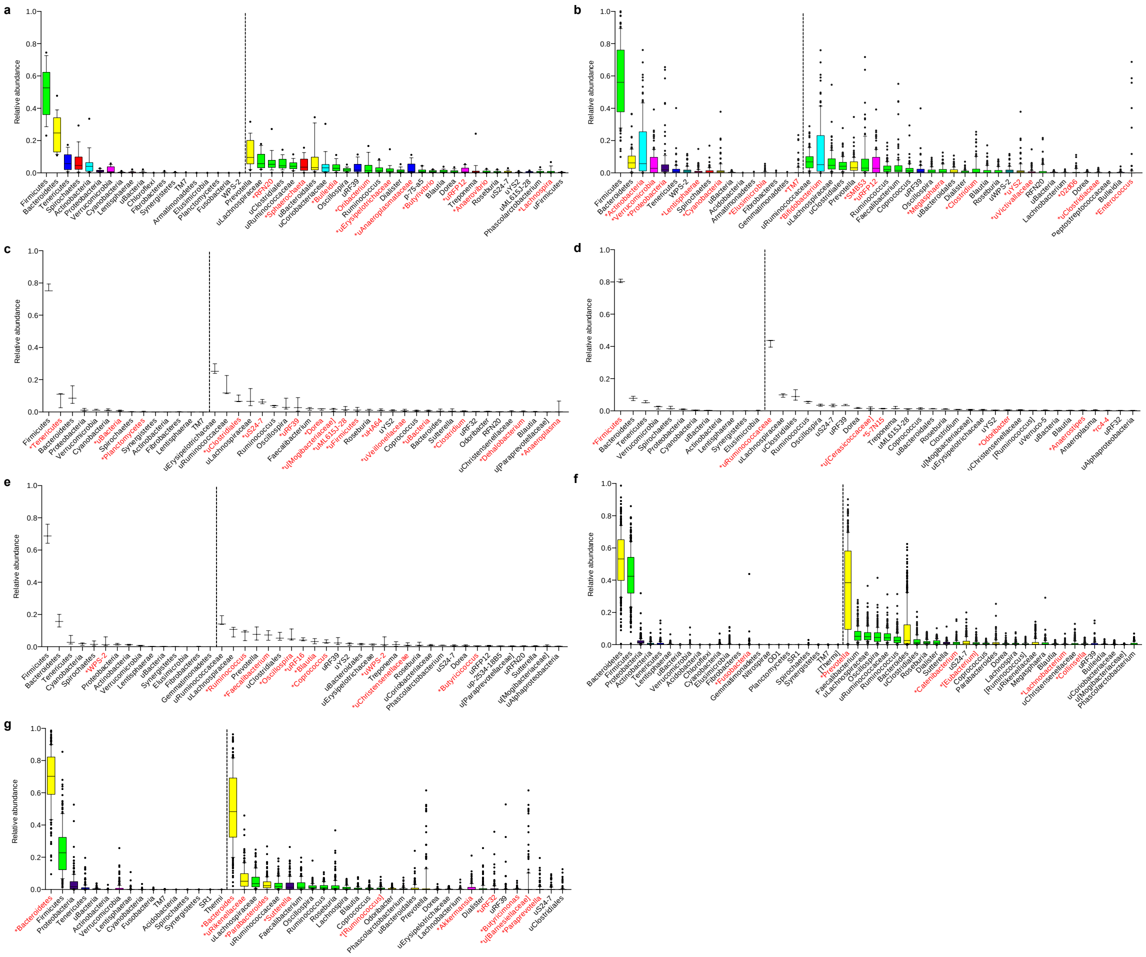

Supplementary Figure 1. Composition of the fecal microbiota of other non-human primates and humans. Box-and-whiskers plots showing the relative abundance distributions of all bacterial phyla (left of dotted line), as well as the 30 most abundant bacterial genera (right of dotted line) in **a)** chimpanzees from the Republic of Congo, **b)** baboons from Kenya, **c)** black-and-white colobi from Uganda, **d)** red colobi from Uganda, **e)** red-tailed guenons from Uganda, **f)** humans from Mongolia, and **g)** humans from the U.S. Phyla and genera labels in red with asterisks indicate bacterial taxa identified by LEfSe as associated with each host type compared to other host types (see **Fig. 2a**). Phyla and genera labels preceded by “u” represent taxonomic groups that could only be classified at higher taxonomic levels.

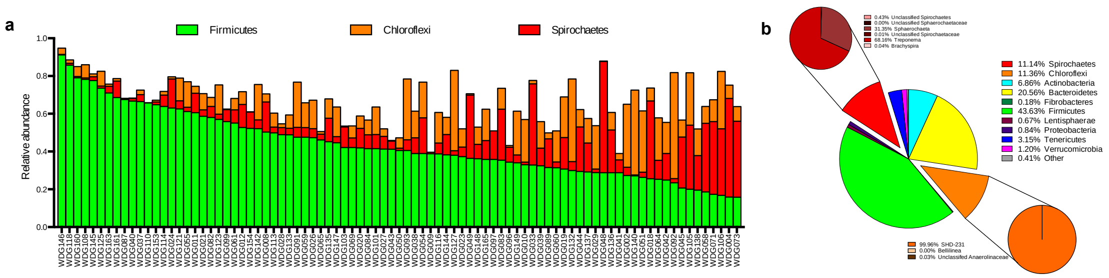

Supplementary Figure 2. Phylum and genus-level composition of the WLG microbiota. **a)** Stacked bar chart showing the relative abundance of Firmicutes, along with Spirochaetes and Chloroflexi in each WLG sample. **b)** Representation of Spirochaetes and Chloroflexi in the microbiota of WLGs. Pie charts showing the mean relative abundance of the Spirochaete and Chloroflexi phyla relative to other phyla in the dataset, as well as the relative abundance distribution of the genera in each phylum.

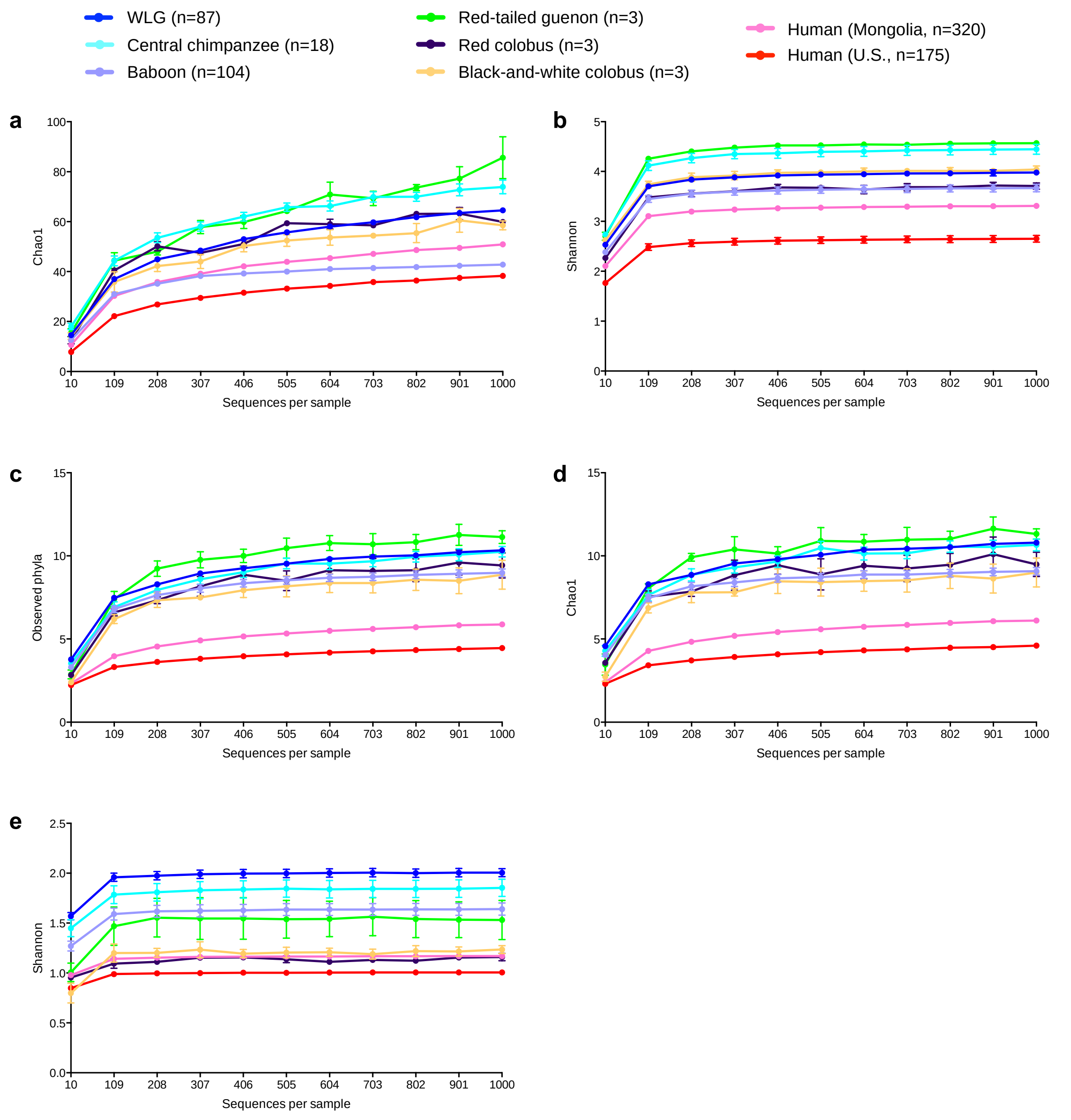

Supplementary Figure 3. Alpha diversity varies among host types and is dependent on taxonomic level. Alpha diversity rarefaction plots based on the genus (**a**-**b**) and phylum (**c**-**e**) using the observed taxa (**c**), Chao1 (**a** and **d**), and Shannon (**b** and **e**) diversity metrics. Mean diversity for each host type is shown with the standard error of the mean.

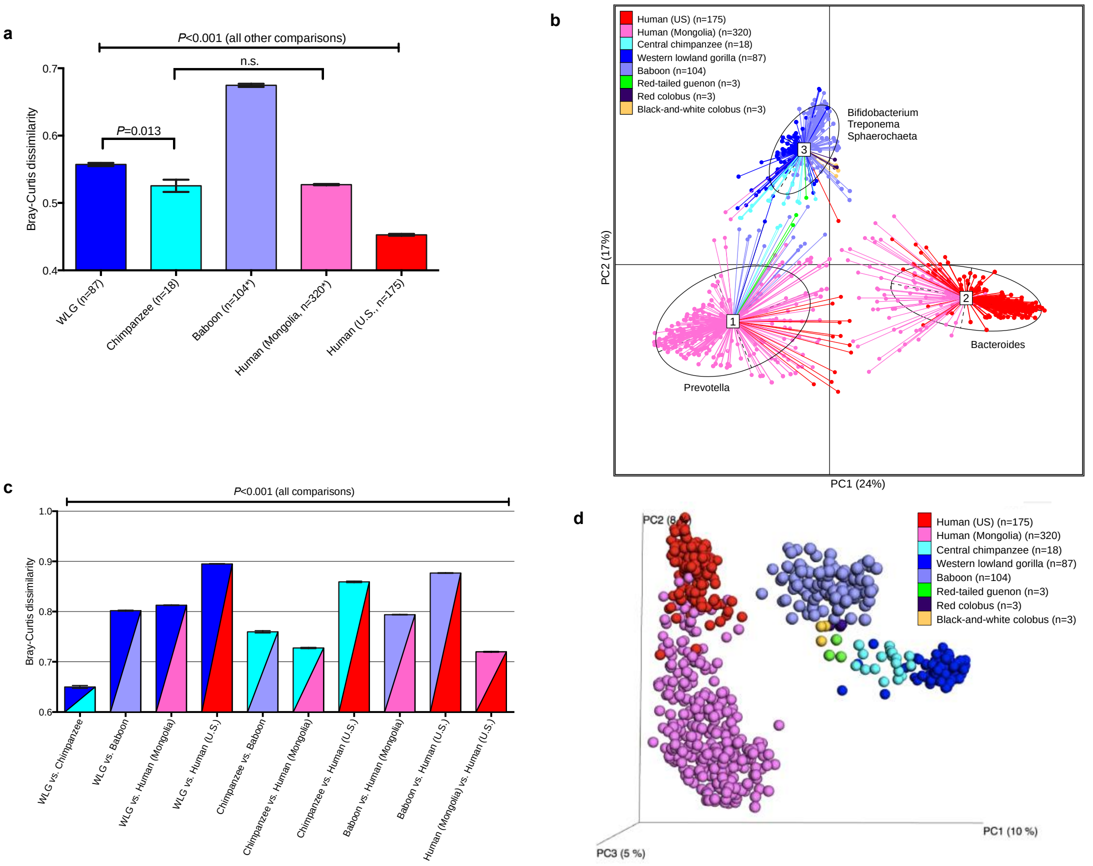

Supplementary Figure 4. Beta diversity varies among host types. **a)** Bar chart showing the mean Bray-Curtis dissimilarity based on the genus-level relative abundance distributions within each host type. *Note that while the Mongolian human and baboon datasets included 320 and 104 samples, respectively, these datasets included longitudinal sampling from the same individuals, and thus only pairwise BC dissimilarities between samples from different individuals were calculated. **b)** Principal coordinate analysis plot showing the three enterotype-like clusters of human and NHP samples identified by PAM based on the rJSD among genus-level relative abundance profiles. Numbered white rectangles indicate the centroid of each cluster, while solid black lines indicate the distance of each sample from the centroid of the cluster. Genera whose relative abundance defines each cluster identified by between class analysis and LEfSe are indicated (see **Supplementary Fig. 5c**). **c)** Bar chart showing the mean Bray-Curtis dissimilarity based on the genus-level relative abundance distributions between samples from different host types. **d)** Principal coordinate analysis plot based on the unweighted UniFrac distance among bacterial 97% OTU-level abundance distributions for each host type. Error bars in **a** and **c** indicate the standard error of the mean. *P* values indicated in **a** and **c** are derived from two-tailed Mann-Whitney tests. n.s., not significant at α=0.05.

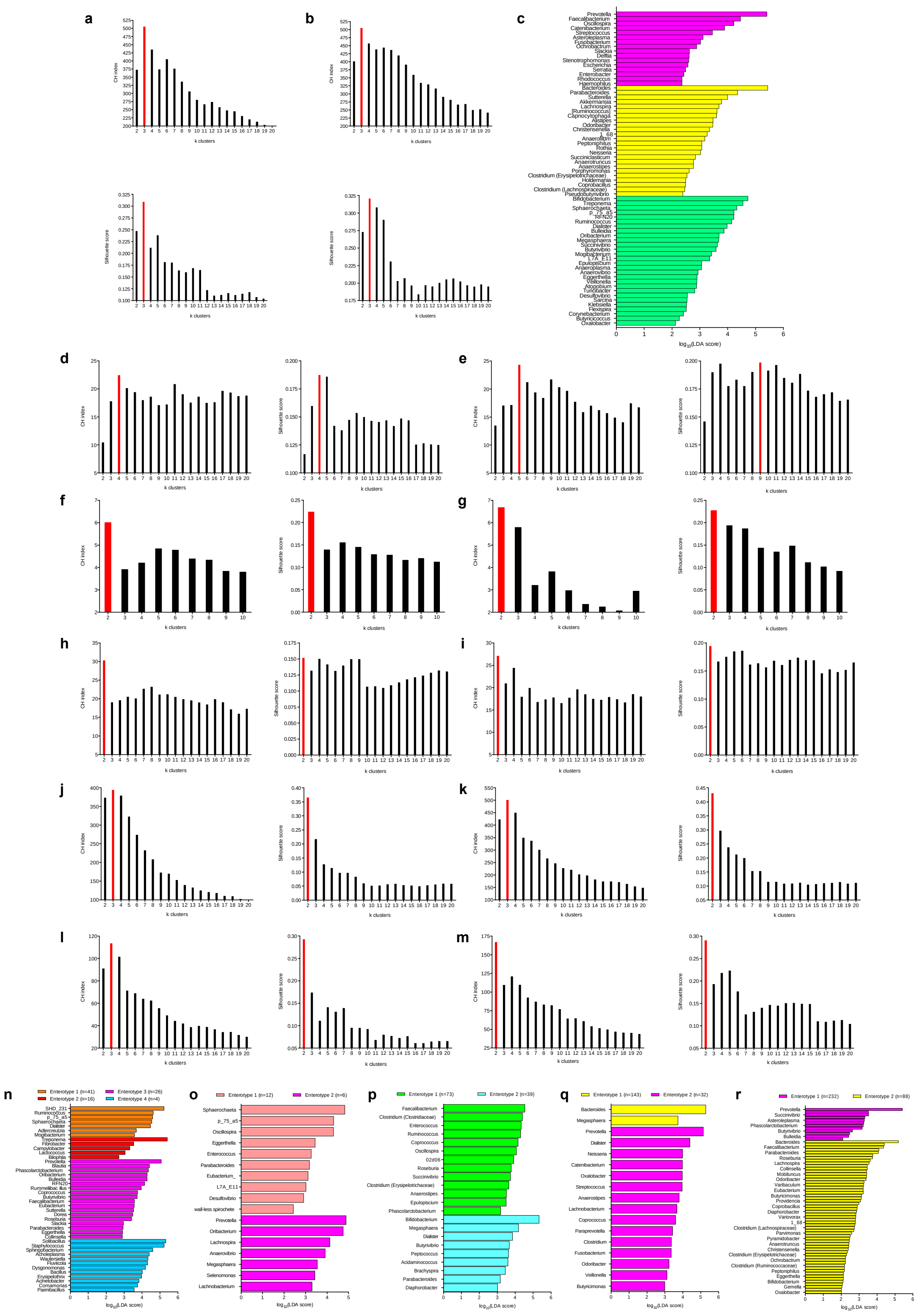

Supplementary Figure 5. Optimal number of clusters (*k*) for PAM analyses and bacterial genera whose relative abundance defines enterotypes of the full dataset, WLGs, chimpanzees, baboons, Mongolian humans, and U.S. humans.

Calińksi-Harabasz (CH) indices and silhouette scores for the full dataset with samples from all host types (**a-b**), the WLG dataset (**d-e**), the chimpanzee dataset (**f-g**), the baboon dataset (**h-i**), the Mongolian human dataset (**j-k**), and the U.S. human dataset (**l-m**) based on rJSD (**a, d, f, h, j,** and **l**) and BC (**b, e, g, i, k,** and **m**) among genus-level relative abundance distributions. For each plot, the *k* with the highest CH or silhouette score is indicated in red. Log-transformed LDA scores derived from LEfSe analysis among samples from the clusters identified by the PAM analyses of **c)** samples from all host types, **n)** WLGs, **o)** chimpanzees, **p)** baboons, **q)** Mongolian humans, and **r)** U.S. humans.

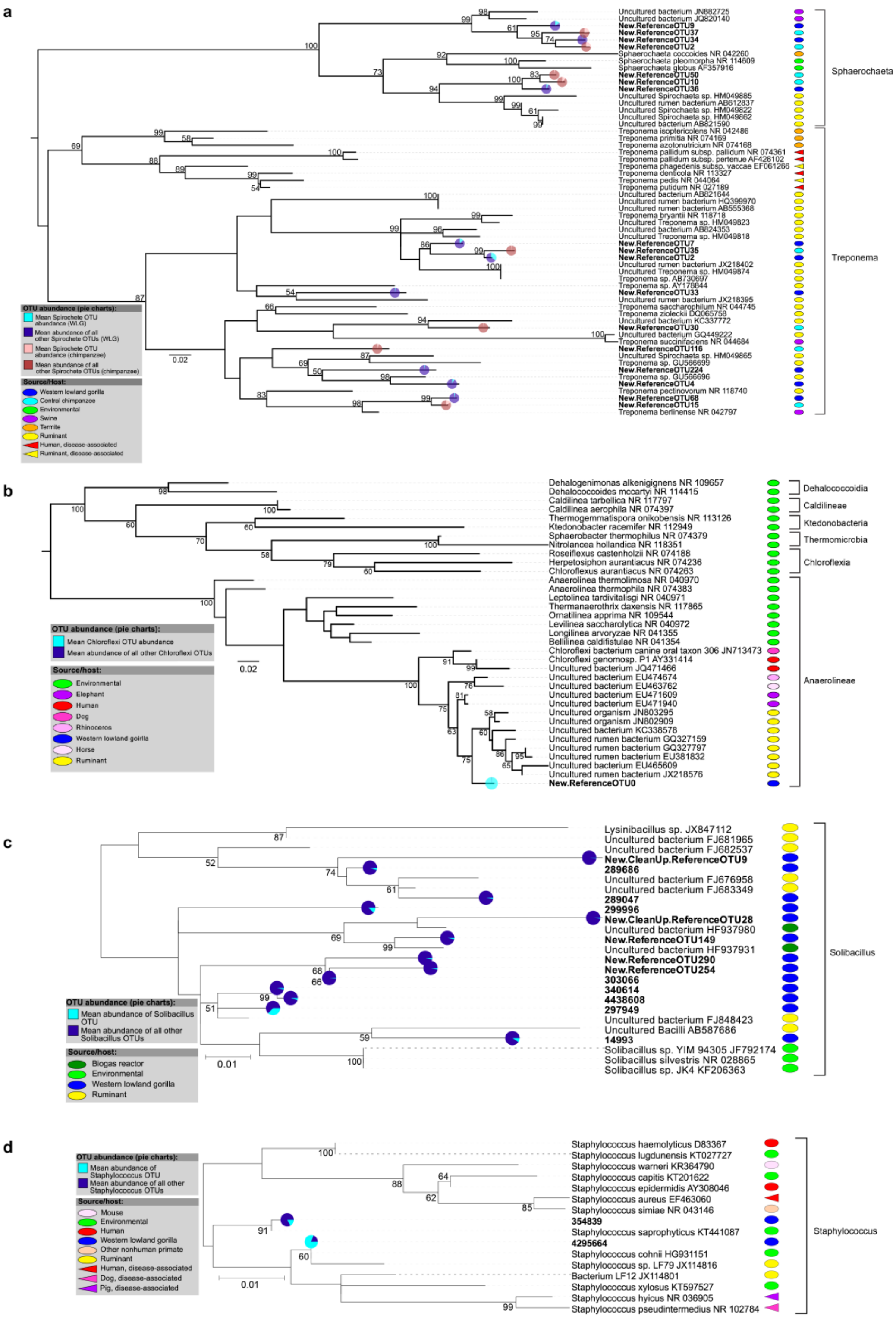

Supplementary Figure 6. Genera whose relative abundance defines unique WLG and chimpanzee enterotypes are most closely related to sequences derived from ruminants. Maximum likelihood trees showing the phylogenetic

relationships among representative sequences of the most abundant WLG **a)** Spirochete (*Treponema* and *Sphaerochaeta*), **b)** Chloroflexi (*SHD-231*), **c)** *Solibacillus*, and **d)** *Staphylococcus* 97% OTUs, as well as the most abundant chimpanzee **a)** Spirochete (*Treponema* and *Sphaerochaeta*) 97% OTUs. OTUs included had the highest mean relative abundance of **a)** Spirochetes, **b)** Chloroflexi, **c)** *Solibacillus*, and **d)** *Staphylococcus* across all WLG (or chimpanzee in **a**) samples and had cumulative mean relative abundance accounting for >90% of all **a)** Spirochete, **b)** Chloroflexi, **c)** *Solibacillus*, and **d)** *Staphylococcus* OTU relative abundance in WLGs (or chimpanzees in **a**). The mean relative abundance of each **a)** Spirochete, **b)** Chloroflexi, **c)** *Solibacillus*, or **d)** *Staphylococcus* OTU compared to the mean relative abundance of all **a)** Spirochete, **b)** Chloroflexi, **c)** *Solibacillus*, or **d)** *Staphylococcus* OTUs from the WLG (and chimpanzee in **a**) dataset is indicated by pie charts. The source or host of each sequence included is indicated by color and shape (see legends). Sequences from GenBank are designated by sequence name and GenBank accession number. Bootstrap values >50% are indicated at the nodes.

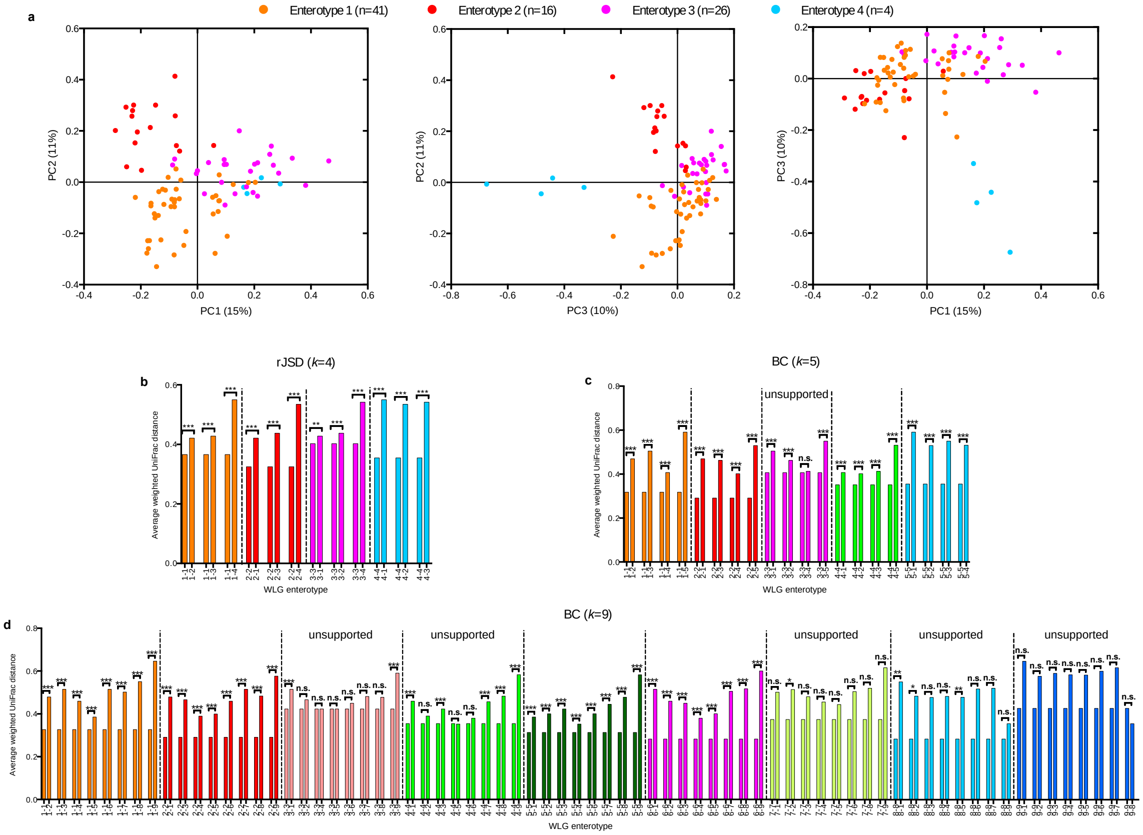

Supplementary Figure 7. BC dissimilarity and weighted UniFrac distance support PAM clustering of WLG samples based on rJSD. **a)** PCoA plots based on BC dissimilarity with sample colors indicating the four WLG clusters identified by PAM clustering based on rJSD. Bar charts showing the average pairwise distances based on weighted UniFrac between WLG samples within **b)** each of the of the four clusters based on rJSD, **c)** each of the five clusters based on BC dissimilarity, and **d)** each of the nine clusters based on BC. Asterisks indicate significance levels determined by a Monte Carlo label permutation approach of comparing within-cluster and between-cluster distances with Bonferroni correction for multiple comparisons (*q<0.05; **q<0.01; ***q<0.001; n.s., not significant). Unsupported clusters are indicated in **c-d**, wherein within-cluster pairwise distances are not significantly different from between-cluster pairwise distances.

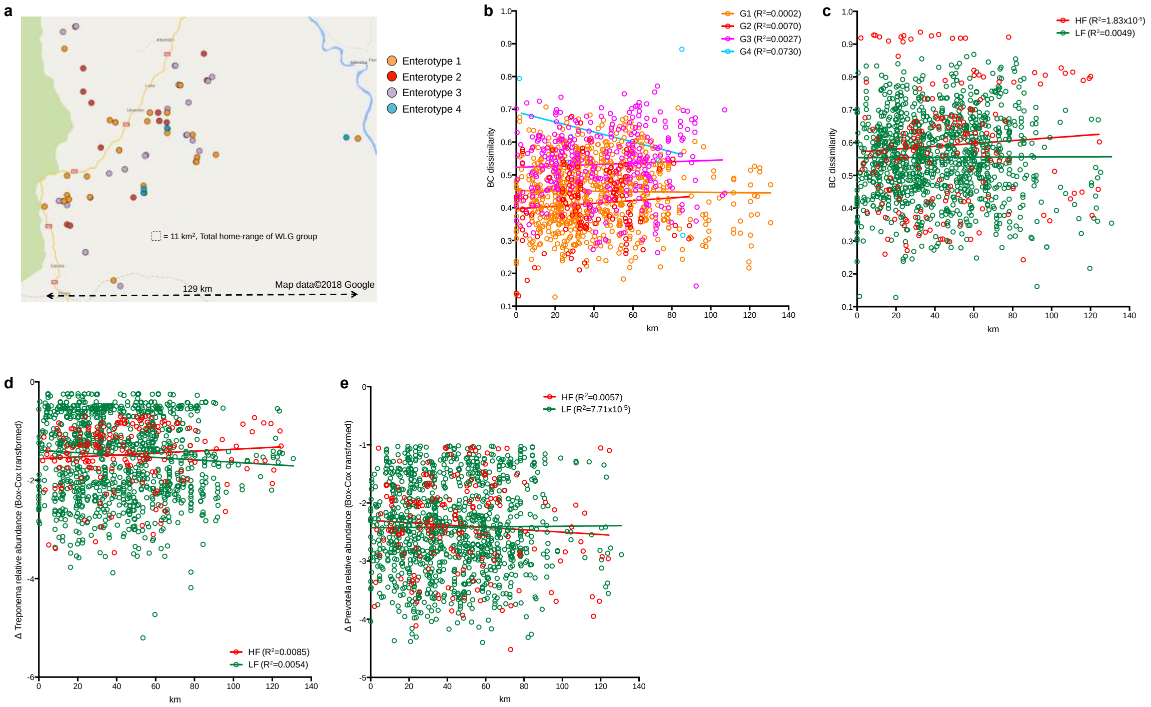

Supplementary Figure 8. WLG enterotypes, high and low frugivory WLG microbiota, and the relative abundance of seasonally fluctuant bacterial genera whose relative abundance defines WLG enterotypes are not associated with geographic distance between samples. **a)** Collection sites of the 87 WLG fecal samples with sample colors indicating the four WLG enterotypes. The map and geolocation for the sites of each fecal sample's collection (colored circles), based on GPS coordinates, was created with Google My Maps and Map data (Map data ©2018 Google). Scatter plots showing the lack of association between pairwise BC dissimilarities and distance between locations of sample collection in **b)** samples from each of the four WLG enterotypes, and **c)** samples from the HF and LF seasons. Scatter plots showing the lack of association between distance between locations of sample collection and change in **d)** *Treponema* and **e)** *Prevotella* relative abundance in samples from the HF and LF seasons.

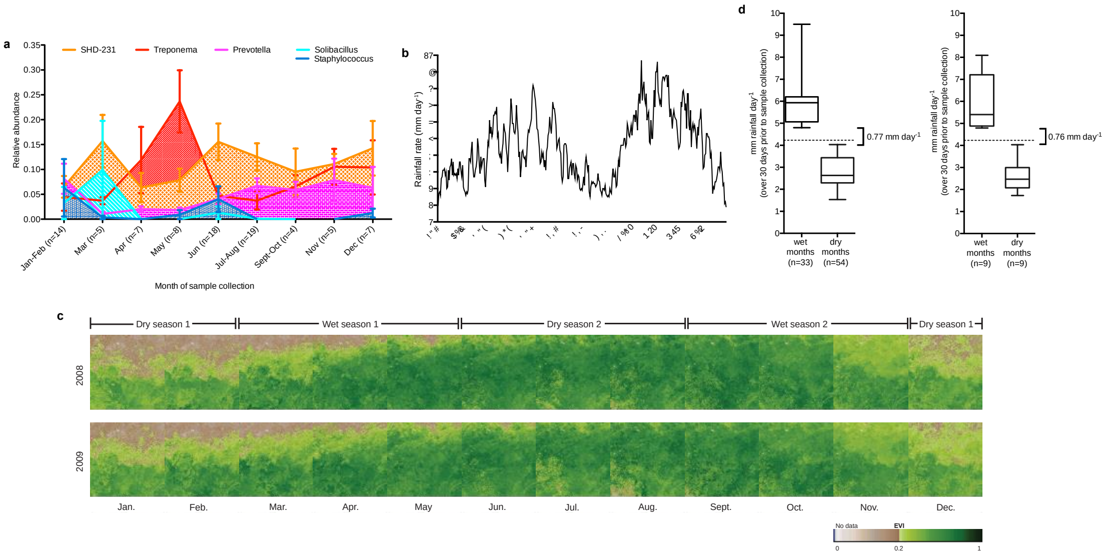

Supplementary Figure 9. Relative abundance of WLG and chimpanzee enterotype-defining taxa may vary temporally, reflecting seasonal variation in rainfall and vegetation. **a)** Area chart showing the mean monthly relative abundance in WLGs of the genera defining enterotype 1 (*SHD-231*), enterotype 2 (*Treponema*), enterotype 3 (*Prevotella*) and enterotype 4 (*Solibacillus* and *Staphylococcus*). Error bars indicate the standard error of the mean. **b)** Line graph showing the average daily rainfall rate (5-day rolling average based on TRMM satellite data) for the Sangha region over a 10-year period (2001-2010). **c)** Sangha region seasonal vegetation. Per-month enhanced vegetation indices (EVI) based on Terra/MODIS satellite data for the region of sample collection from 2008-2009. **d)** Box-and-whiskers plots showing the distribution of the total average daily rainfall over the 30 days prior to sample collection for WLG (left) and chimpanzee (right) samples collected after wet periods (>4.23 mm day^{-1}) and after dry periods (<4.23 mm day^{-1}). Note the difference (0.77 mm day^{-1} and 0.76 mm day^{-1} for WLG and chimpanzee samples, respectively) in total rainfall between the sample with the lowest total rainfall in the wet month group and the sample with the highest total rainfall in the dry month group.

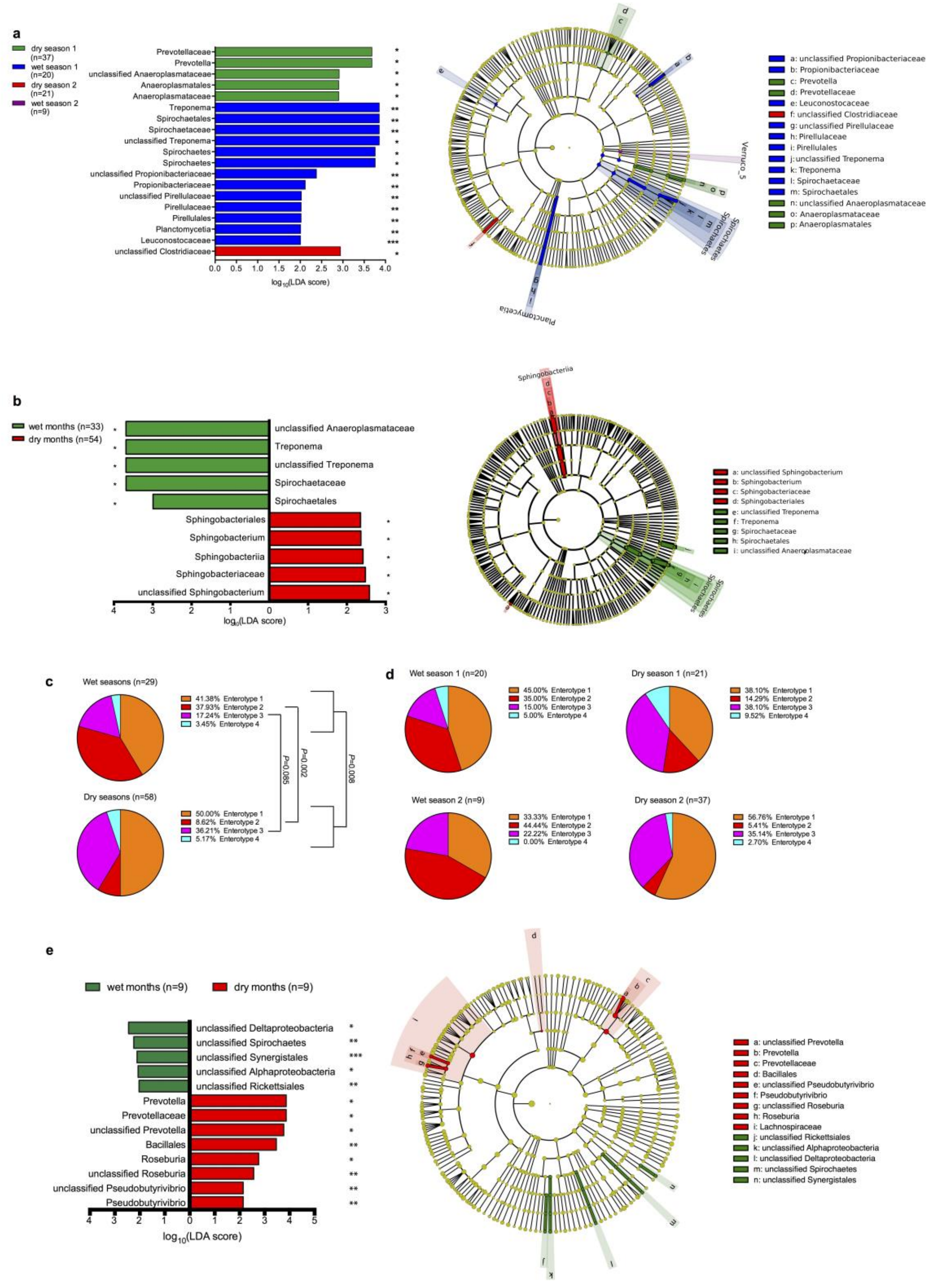

Supplementary Figure 10. The microbiota and enterotypes of WLGs and chimpanzees vary among wet and dry seasons. **a)** Bar chart showing the log-transformed LDA scores and cladogram showing the phylogenetic relationships of bacterial taxa found to be significantly associated with season of sample

collection in WLG samples by LEfSe. **b)** Bar chart showing the log-transformed LDA scores and cladogram showing the phylogenetic relationships of bacterial taxa found to be significantly associated with wet or dry months in WLG samples by LEfSe. **c)** Pie charts showing the proportion of WLG samples from each enterotype in wet (wet season 1 and wet season 2) and dry (dry season 1 and dry season 2) seasons. Two-tailed *P* values from Chi-square test (all enterotypes in dry seasons vs. wet seasons) and Fisher's exact tests (enterotype 2 in dry seasons vs. wet seasons, enterotype 3 in dry seasons vs. wet seasons) are indicated. **d)** Pie charts showing the proportion of WLG samples from each enterotype in each of the four seasons. **e)** Bar chart showing the log-transformed LDA scores and cladogram showing the phylogenetic relationships of bacterial taxa found to be significantly associated with wet or dry months in chimpanzee samples by LEfSe. Wet and dry seasons in **a** and **d** were defined by total monthly rainfall patterns averaged over a 10-year period (2001-2010, see **Fig. 4a**). Wet and dry months in **b** and **e** were defined by average daily rainfall for the 30 days prior to the date of sample collection (>4.23 mm day^{-1} for wet months, <4.23 mm day^{-1} for dry months, see **Supplementary Fig. 9d**). Asterisks in **a**, **b**, and **e** indicate that Box-Cox-transformed bacterial relative abundance was significantly predicted by season of sample collection in ANOVA analyses (**a**) or by average daily rainfall for the 30 days prior to sample collection in ANCOVA analyses (**b** and **e**), adjusting for the effects of potentially confounding variables. **P*<0.05, ***P*<0.01, ****P*<0.001.

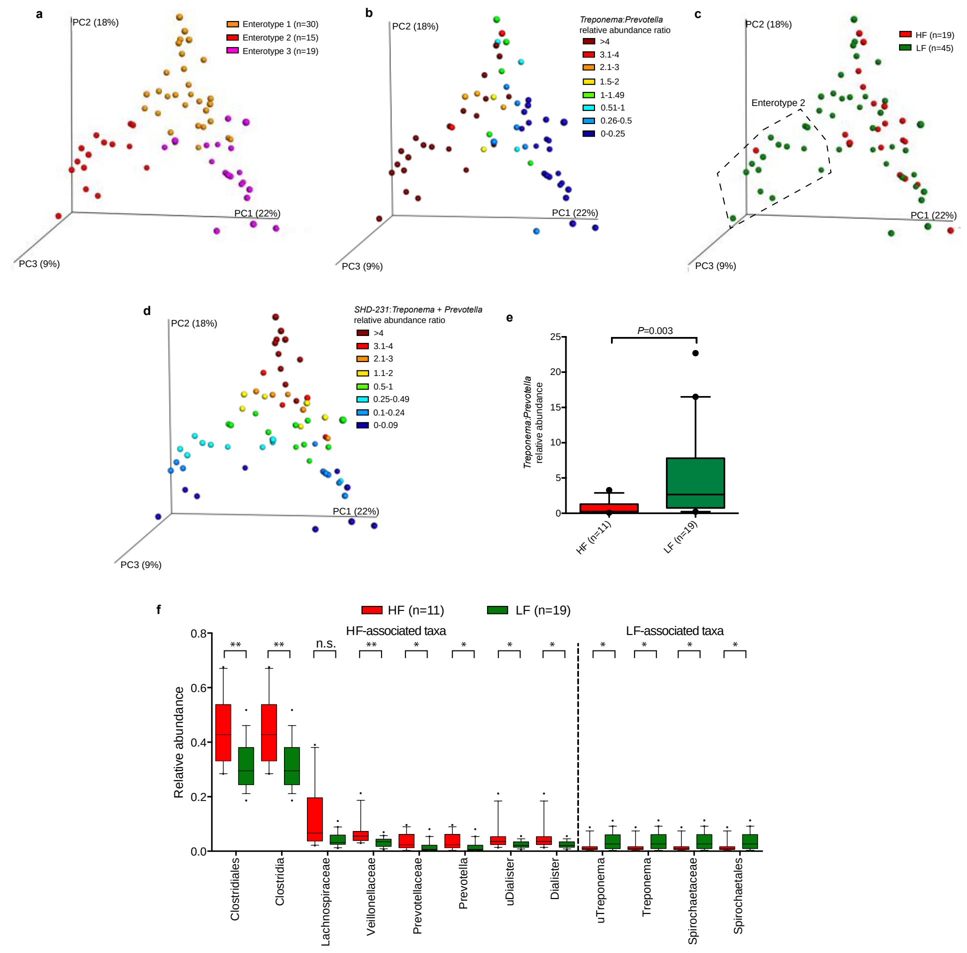

Supplementary Figure 11. Samples from WLG enterotypes 1-3 cluster based on gradients of *SHD-231*, *Treponema*, and *Prevotella* relative abundance, the latter two of which are associated with the low frugivory (LF) and high frugivory (HF) seasons. Principal coordinates analysis plots based on Bray-Curtis dissimilarity among genus-level abundance distributions of samples from WLG enterotypes 1-3 that were collected during HF or LF periods colored by **a)** WLG enterotype, **b)** the ratio of *Treponema* relative abundance to *Prevotella* relative abundance, **c)** the season (HF or LF) in which the sample was collected, and **d)** the ratio of *SHD-231* relative abundance to the cumulative *Treponema* and *Prevotella* relative abundance. Box-and-whiskers plots showing **e)** the ratio of *Treponema* relative abundance to *Prevotella* relative abundance in WLG enterotype 1 samples collected from HF periods compared to those collected during LF periods and **f)** the relative abundance of the HF- and LF-associated bacterial taxa (from LEfSe analysis of the all WLG samples) in WLG enterotype 1 samples collected during HF (red) and LF (green) seasons. Two-tailed *P*-values or significance levels based on two-tailed *P* values from Mann-Whitney tests, where *P<0.05 and **P<0.01, are indicated in **e** and **f**. n.s., not significant at α=0.05.

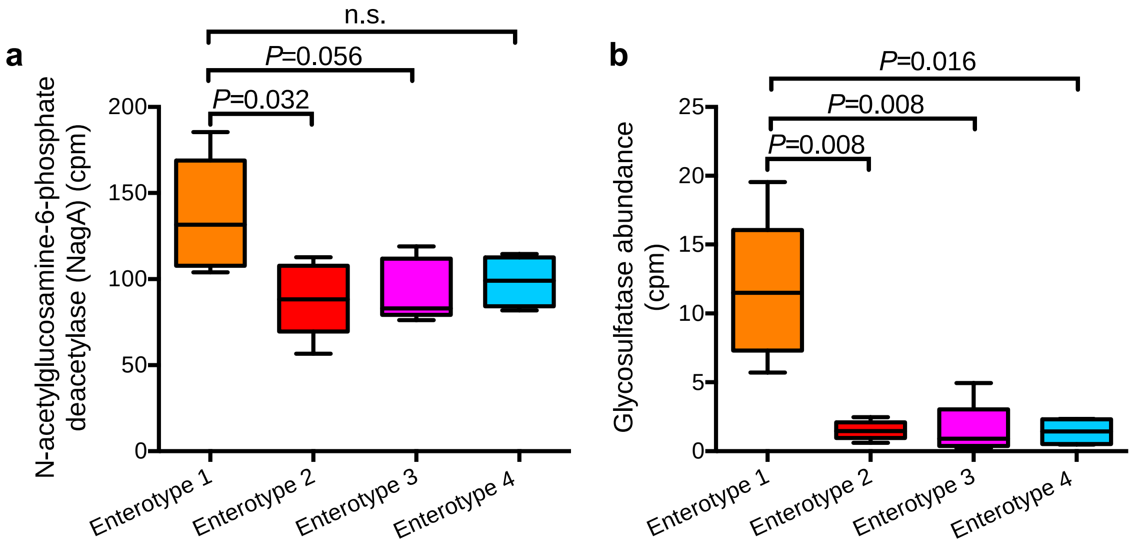

Supplementary Figure 12. Genes coding for carbohydrate utilization enzymes enriched in the Chloroflexi-abundant WLG enterotype 1. Box-and-whiskers plots showing the relative abundance of **a)** N-acetylglucosamine-6-phospohate deacetylase (NagA) and **b)** glycosulfatase (identified by HUMAnN2 analysis) in each of the WLG enterotypes 1 (n=5), 2 (n=5), 3 (n=5) and 4 (n=4). Two-tailed *P* values from Mann-Whitney tests are indicated. n.s., not significant at α=0.05.

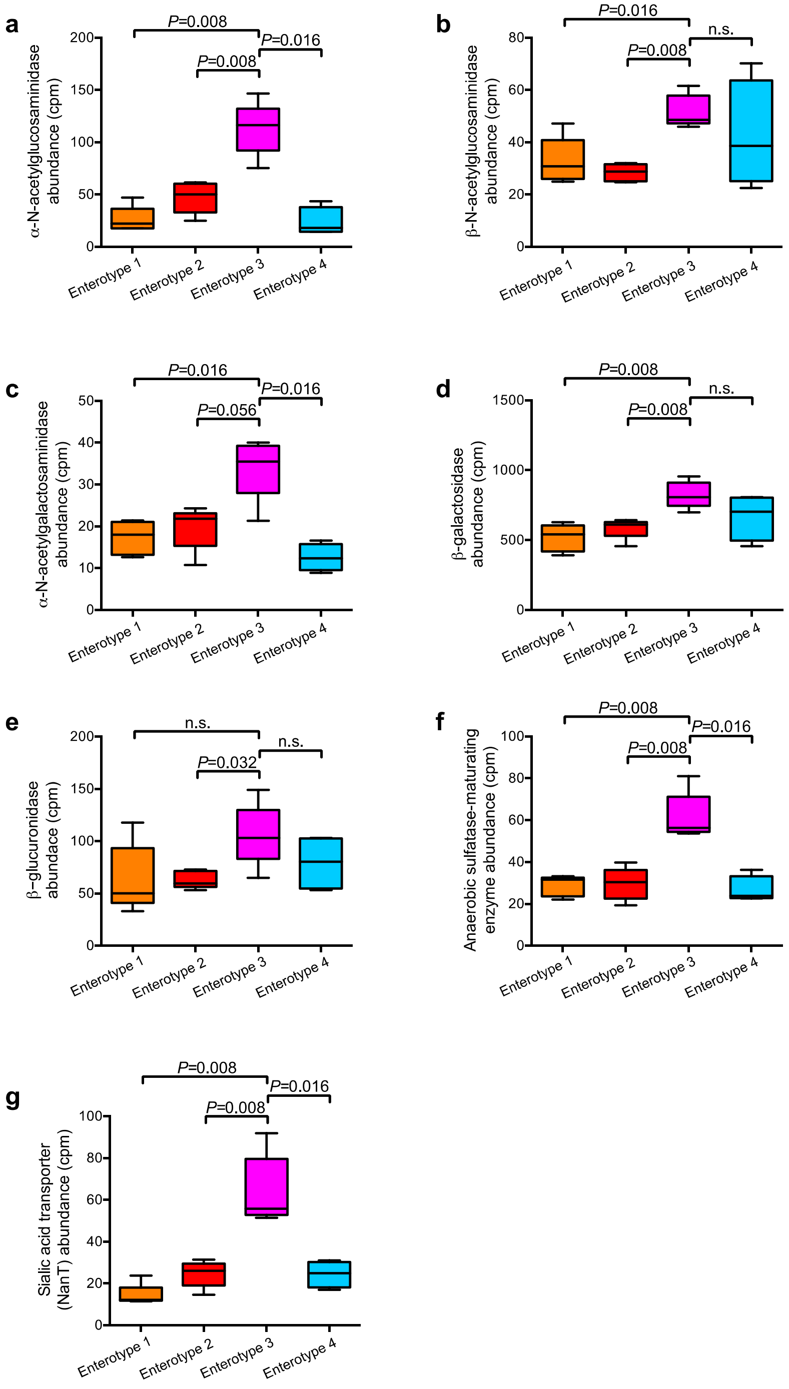

Supplementary Figure 13. Genes encoding enzymes involved in other mucus degradative pathways are elevated in *Prevotella*-abundant enterotype 3. **a-g)** Box-and-whiskers plots showing the relative abundance of additional genes (identified by HUMAnN2 analysis) encoding enzymes involved in mucus degradation in samples from WLG enterotypes 1 (n=5), 2 (n=5), 3 (n=5) and 4 (n=4). Two-tailed *P* values from Mann-Whitney tests are indicated**.** n.s., not significant at α=0.05.

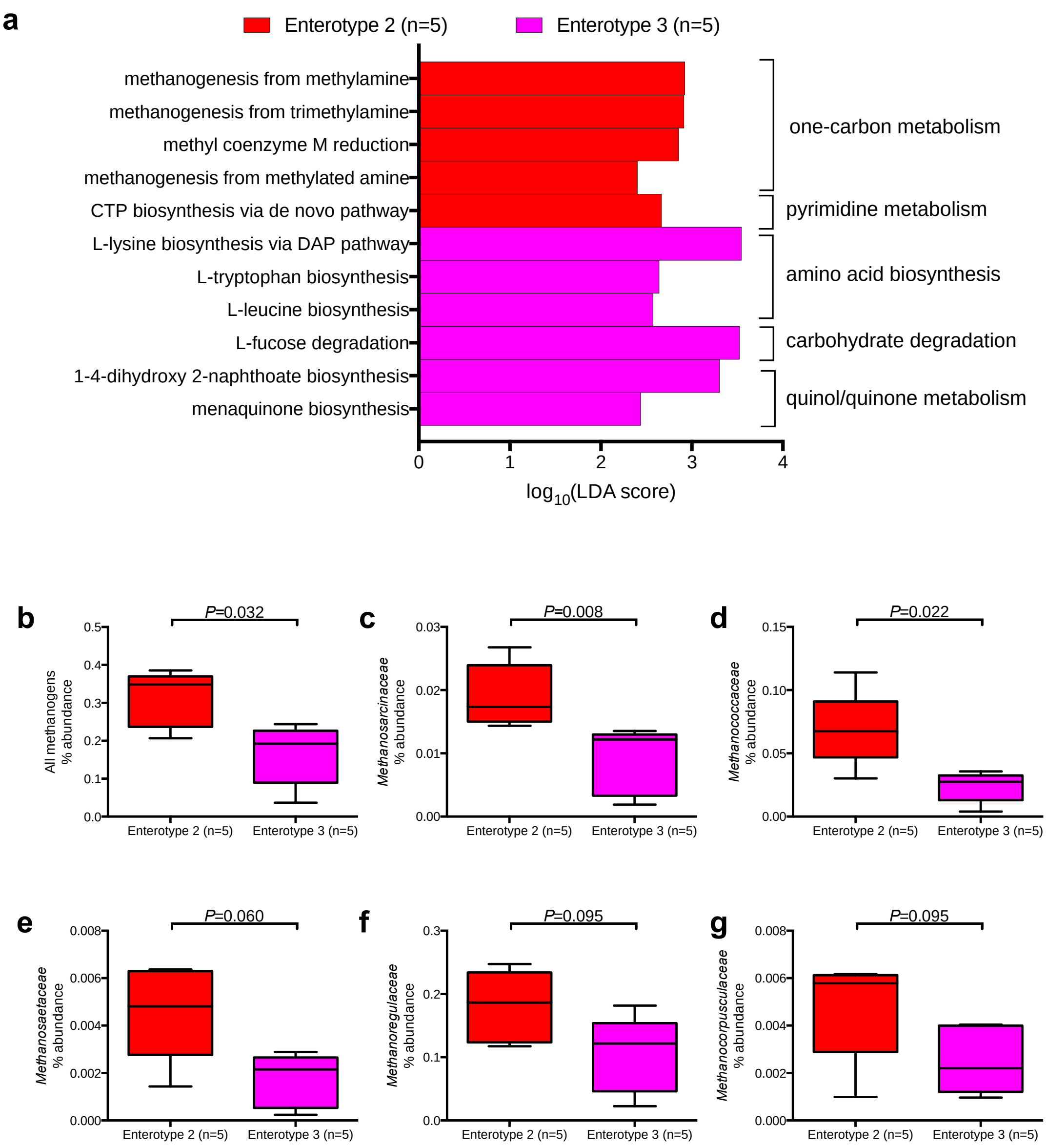

Supplementary Figure 14. Metagenomic comparison of the seasonally fluctuant enterotypes 2 and 3 reveals enrichment in methanogenesis functional pathways and methanogenic archaea in enterotype 2. **a)** Bar chart showing LEfSe-identified log-transformed LDA scores for functional pathways (from HUMAnN2 analysis) that distinguish WLG enterotype 2 (*Treponema*-abundant) from WLG enterotype 3 (*Prevotella*-abundant) with the superpathways to which the pathways belong indicated on the right. Only pathways associated with significant superpathways and with a log-transformed LDA score $\geq$3 are shown. **b-g)** Relative abundance of all methanogenic archaea **(b)** and select families of methanogenic archaea **(c-g)** in enterotype 2 and enterotype 3 samples based on MetaPhlAn2 analysis of shotgun metagenomic sequences. Two-tailed *P* values from Mann-Whitney tests are indicated in **b-g**.

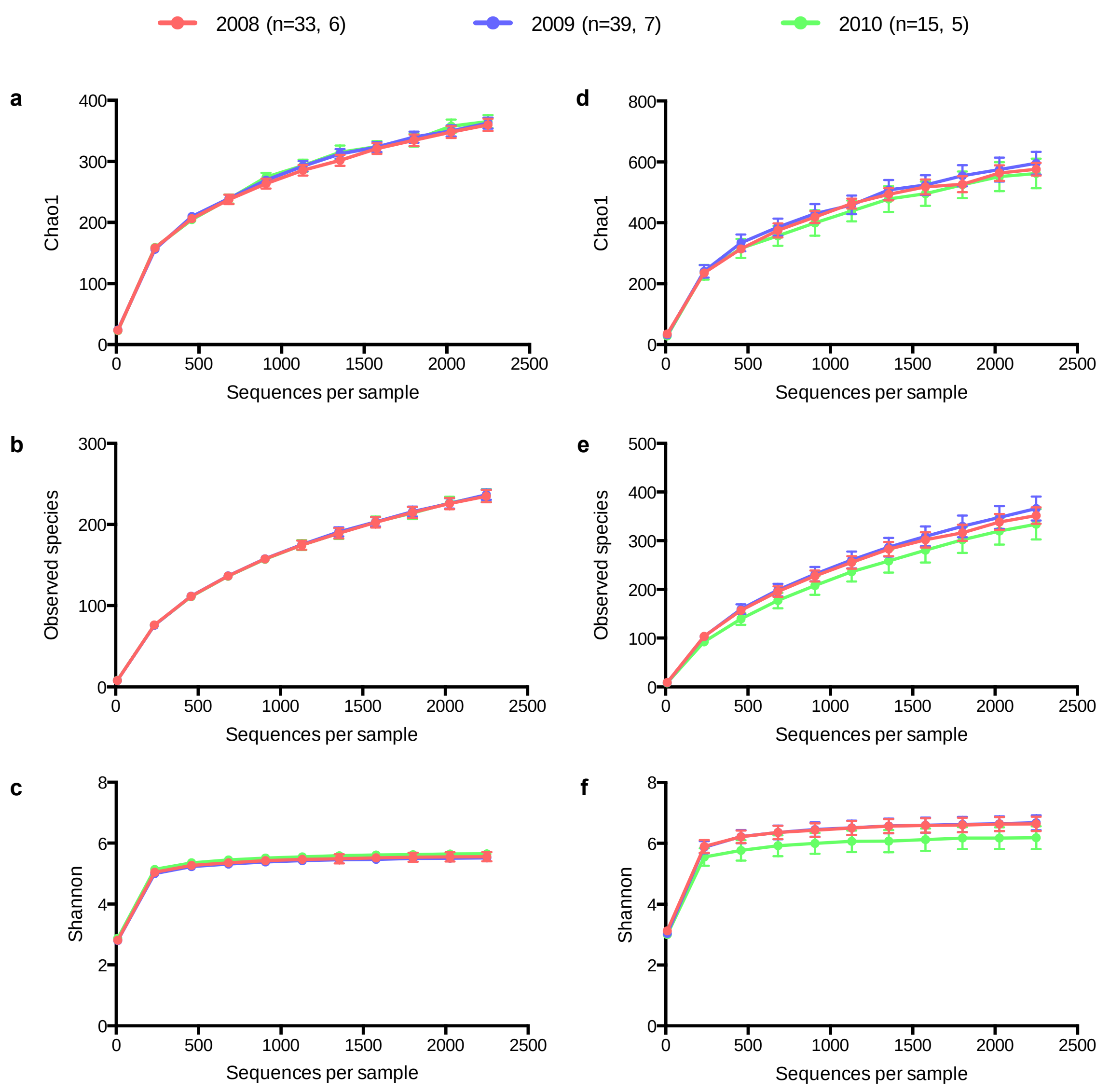

Supplementary Figure 15. Alpha diversity among WLG and chimpanzee samples does not vary by year of sample collection. Alpha diversity rarefaction plots for OTUs (97% clustering) from WLG (**a-c**) and chimpanzee (**d-f**) samples collected in each year based on the chao1 (**a, d**), observed species (**b, e**), and Shannon diversity (**c, f**) metrics. Mean diversity and standard error of the mean are indicated in rarefaction plots.

SUPPLEMENTARY REFERENCES

1 Hooper, L. V. & Gordon, J. I. Commensal host-bacterial relationships in the gut. *Science* **292**, 1115-1118 (2001).

2 Arumugam, M. *et al.* Enterotypes of the human gut microbiome. *Nature* **473**, 174-180, doi:10.1038/nature09944 (2011).

3 Wu, G. D. *et al.* Linking long-term dietary patterns with gut microbial enterotypes. *Science* **334**, 105-108, doi:10.1126/science.1208344 (2011).

4 Zhang, J. C. *et al.* Mongolians core gut microbiota and its correlation with seasonal dietary changes. *Sci Rep-Uk* **4**, doi:Artn 5001
10.1038/Srep05001 (2014).

5 Moeller, A. H. *et al.* Sympatric chimpanzees and gorillas harbor convergent gut microbial communities. *Genome research* **23**, 1715-1720, doi:10.1101/gr.154773.113 (2013).

6 Koren, O. *et al.* A guide to enterotypes across the human body: meta-analysis of microbial community structures in human microbiome datasets. *PLoS computational biology* **9**, e1002863, doi:10.1371/journal.pcbi.1002863 (2013).

7 Arumugam, M. *et al.* Enterotypes of the human gut microbiome. *Nature* **473**, 174-180, doi:Doi 10.1038/Nature09944 (2011).

8 Moeller, A. H. *et al.* Chimpanzees and humans harbour compositionally similar gut enterotypes. *Nature communications* **3**, 1179, doi:10.1038/ncomms2159 (2012).

9 Ren, T., Grieneisen, L. E., Alberts, S. C., Archie, E. A. & Wu, M. Development, diet and dynamism: longitudinal and cross-sectional predictors of gut microbial communities in wild baboons. *Environmental microbiology* **18**, 1312-1325, doi:10.1111/1462-2920.12852 (2016).

10 Costea, P. I. *et al.* Enterotypes in the landscape of gut microbial community composition. *Nat Microbiol* **3**, 8-16, doi:10.1038/s41564-017-0072-8 (2018).

11 Sutcliffe, I. C. Cell envelope architecture in the Chloroflexi: a shifting frontline in a phylogenetic turf war. *Environmental microbiology* **13**, 279-282, doi:10.1111/j.1462-2920.2010.02339.x (2011).

12 Denef, V. J., Mueller, R. S., Chiang, E., Liebig, J. R. & Vanderploeg, H. A. Chloroflexi CL500-11 Populations That Predominate Deep-Lake Hypolimnion Bacterioplankton Rely on Nitrogen-Rich Dissolved Organic Matter Metabolism and C1 Compound Oxidation. *Applied and environmental microbiology* **82**, 1423-1432, doi:10.1128/AEM.03014-15 (2015).

13 Hug, L. A. *et al.* Community genomic analyses constrain the distribution of metabolic traits across the Chloroflexi phylum and indicate roles in sediment carbon cycling. *Microbiome* **1**, 22, doi:10.1186/2049-2618-1-22 (2013).

14 Campbell, A. G. *et al.* Diversity and genomic insights into the uncultured Chloroflexi from the human microbiota. *Environmental microbiology* **16**, 2635-2643, doi:10.1111/1462-2920.12461 (2014).

15 Samuel, G. & Reeves, P. Biosynthesis of O-antigens: genes and pathways involved in nucleotide sugar precursor synthesis and O-antigen assembly. *Carbohyd Res* **338**, 2503-2519, doi:10.1016/j.carres.2003.07.009 (2003).
16 Shinkai, T., Ueki, T. & Kobayashi, Y. Detection and identification of rumen bacteria constituting a fibrolytic consortium dominated by Fibrobacter succinogenes. *Animal science journal = Nihon chikusan Gakkaiho* **81**, 72-79, doi:10.1111/j.1740-0929.2009.00698.x (2010).
17 Wright, D. P., Knight, C. G., Parkar, S. G., Christie, D. L. & Roberton, A. M. Cloning of a mucin-desulfating sulfatase gene from Prevotella strain RS2 and its expression using a Bacteroides recombinant system. *Journal of bacteriology* **182**, 3002-3007 (2000).
18 Wright, D. P., Rosendale, D. I. & Robertson, A. M. Prevotella enzymes involved in mucin oligosaccharide degradation and evidence for a small operon of genes expressed during growth on mucin. *FEMS microbiology letters* **190**, 73-79 (2000).
19 Benjdia, A., Martens, E. C., Gordon, J. I. & Berteau, O. Sulfatases and a radical S-adenosyl-L-methionine (AdoMet) enzyme are key for mucosal foraging and fitness of the prominent human gut symbiont, Bacteroides thetaiotaomicron. *The Journal of biological chemistry* **286**, 25973-25982, doi:10.1074/jbc.M111.228841 (2011).
20 Fuchs, G., Boll, M. & Heider, J. Microbial degradation of aromatic compounds - from one strategy to four. *Nat Rev Microbiol* **9**, 803-816, doi:10.1038/nrmicro2652 (2011).
21 Iason, G. R. & Villalba, J. J. Behavioral strategies of mammal herbivores against plant secondary metabolites: the avoidance-tolerance continuum. *Journal of chemical ecology* **32**, 1115-1132, doi:10.1007/s10886-006-9075-2 (2006).
22 Freeland, W. J. & Janzen, D. H. Strategies in Herbivory by Mammals - Role of Plant Secondary Compounds. *Am Nat* **108**, 269-289, doi:Doi 10.1086/282907 (1974).
23 Kimball, B. & FD, P. Chemical Defense and Mammalian Herbivores. *USDA National Wildlife Research Center-Staff Publications* (2003).
24 Carmona, M. *et al.* Anaerobic catabolism of aromatic compounds: a genetic and genomic view. *Microbiology and molecular biology reviews : MMBR* **73**, 71-133, doi:10.1128/MMBR.00021-08 (2009).
25 Rogers, M. E. *et al.* Western gorilla diet: a synthesis from six sites. *American journal of primatology* **64**, 173-192, doi:10.1002/ajp.20071 (2004).
26 Lin, C. H. & Pan, T. M. Assessing the digestion of a genetically modified tomato (Solanum lycopersicum) R8 DNA in simulated gastric fluid using event-specific real-time PCR. *Eur Food Res Technol* **232**, 1061-1067, doi:10.1007/s00217-011-1479-8 (2011).
27 Vieira-Silva, S. *et al.* Species-function relationships shape ecological properties of the human gut microbiome. *Nat Microbiol* **1**, doi:Artn 16088
10.1038/Nmicrobiol.2016.88 (2016).

28 Tailford, L. E., Crost, E. H., Kavanaugh, D. & Juge, N. Mucin glycan foraging in the human gut microbiome. *Frontiers in genetics* **6**, 81, doi:10.3389/fgene.2015.00081 (2015).
29 Masi, S. *et al.* Unusual feeding behavior in wild great apes, a window to understand origins of self-medication in humans: role of sociality and physiology on learning process. *Physiology & behavior* **105**, 337-349, doi:10.1016/j.physbeh.2011.08.012 (2012).
30 Forbey, J. S. *et al.* Exploitation of secondary metabolites by animals: A response to homeostatic challenges. *Integrative and comparative biology* **49**, 314-328, doi:10.1093/icb/icp046 (2009).
31 Calvert, J. J. Food selection by western gorillas (G.g. gorilla) in relation to food chemistry. *Oecologia* **65**, 236-246, doi:10.1007/BF00379223 (1985).
32 Mosele, J. I., Macia, A. & Motilva, M. J. Metabolic and Microbial Modulation of the Large Intestine Ecosystem by Non-Absorbed Diet Phenolic Compounds: A Review. *Molecules* **20**, 17429-17468, doi:10.3390/molecules200917429 (2015).
33 Duda-Chodak, A., Tarko, T., Satora, P. & Sroka, P. Interaction of dietary compounds, especially polyphenols, with the intestinal microbiota: a review. *Eur J Nutr* **54**, 325-341, doi:10.1007/s00394-015-0852-y (2015).
34 Smith, A. H., Zoetendal, E. & Mackie, R. I. Bacterial mechanisms to overcome inhibitory effects of dietary tannins. *Microb Ecol* **50**, 197-205, doi:10.1007/s00248-004-0180-x (2005).
