# Supplementary material for: Gut microbiomes of wild great apes fluctuate seasonally in response to diet
Source: Nat Commun. 2018 May 3;9:1786. doi: 10.1038/s41467-018-04204-w (PMC5934369; doi:10.1038/s41467-018-04204-w)
Supplement: Supplementary file 3 — Description of Additional Supplementary Information [file 41467_2018_4204_MOESM3_ESM.pdf]

**Description of Additional Supplementary Files**

File Name: Supplementary Data 1
Description: Microsatellite analysis of WLG and chimpanzee fecal samples used in this study.

File Name: Supplementary Data 2
Description: 97% OTU table of the WLG microbiota.

File Name: Supplementary Data 3
Description: 97% OTU table of the chimpanzee microbiota.

File Name: Supplementary Data 4
Description: Table for Chloroflexi, *Treponema*, and *Sphaerochaeta* prevalence and relative abundance in primate datasets.

File Name: Supplementary Data 5
Description: Table showing LEfSe analysis of all bacterial taxa distinguishing primate groups.

File Name: Supplementary Data 6
Description: Table showing LEfSe analysis of bacterial functional metabolic superpathways and pathways distinguishing the four WLG enterotype groups.

File Name: Supplementary Data 7
Description: Table showing LEfSe analysis of bacterial functional metabolic superpathways and pathways that most distinguish seasonally fluctuant WLG enterotype 2 compared to enterotype 3 samples.

File Name: Supplementary Data 8
Description: Table showing percent abundance of Archaea in individual WLG samples from each of the four enterotype groups based on shotgun metagenomic sequencing.

File Name: Supplementary Data 9
Description: Table of plants identified in WLG fecal samples based on shotgun metagenomic sequencing analysis.
