# Supplementary material for: Gut microbiomes of wild great apes fluctuate seasonally in response to diet
Source: Nat Commun. 2018 May 3;9:1786. doi: 10.1038/s41467-018-04204-w (PMC5934369; doi:10.1038/s41467-018-04204-w)
Supplement: Supplementary file 9 — Supplementary Data 6 [file 41467_2018_4204_MOESM9_ESM.docx]

**Supplementary Data 6.** Unipathway superpathways and pathways (predicted by HUMAnN2) identified as associated with WLG SHD-231-abundant enterotype 1 (orange), WLG Treponema-abundant enterotype 2 (red), WLG Prevotella-abundant enterotype 3 (pink), or WLG Solibacillus/Staphylococcus-abundant enterotype 4 (blue) samples by LEfSe analysis of the 19 WLG samples selected for shotgun metagenomics sequencing.

| **Superpathway** | **LDA score** | **Pathway** | **LDA score** |
| --- | --- | --- | --- |
| Amine and polyamine biosynthesis | - | putrescine biosynthesis via L-ornithine pathway | 2.55 |
|  |  | S-adenosylmethioninamine biosynthesis | 3.38 |
| Amino acid biosynthesis | - | L-arginine biosynthesis | 3.52 |
|  |  | L-homocysteine biosynthesis | 2.92 |
|  |  | L-serine biosynthesis | 2.61 |
| Amino acid degradation | - | L-kynurenine degradation | 2.04 |
|  |  | L-tryptophan degradation via pyruvate pathway | 2.60 |
| Antibiotic biosynthesis | - | novobiocin biosynthesis | 2.90 |
| Bacterial outer membrane biogenesis | 3.16 | LPS-O-antigen biosynthesis | 3.20 |
| Capsule biogenesis | 2.56 | capsule polysaccharide biosynthesis | 2.52 |
| Carbohydrate acid metabolism | - | D-glucarate degradation | 2.83 |
| Carbohydrate biosynthesis | 3.63 | Calvin cycle | 2.42 |
|  |  | dTDP L-rhamnose biosynthesis | 3.54 |
| Carbohydrate degradation | - | L-arabinose degradation via L-arabinitol | 2.16 |
|  |  | pentose phosphate pathway | 2.91 |
| Carbohydrate metabolism | - | D-galactose 6-phosphate degradation | 2.61 |
|  |  | D-sorbitol biosynthesis | 2.03 |
|  |  | D-tagatose 6-phosphate degradation | 2.54 |
|  |  | fructose metabolism | 2.51 |
|  |  | L-fucose metabolism | 2.84 |
|  |  | nucleotide sugar metabolism | 2.54 |
| Cell wall biogenesis | 3.20 | cell wall polysaccharide biosynthesis | 2.41 |
|  |  | poly(glucopyranosyl N-acetylgalactosamine 1-phosphate) teichoic acid biosynthesis | 2.88 |
|  |  | poly(glycerol phosphate) teichoic acid biosynthesis | 2.77 |
| Cofactor biosynthesis | - | adenosylcobalamin biosynthesis | 3.67 |
|  |  | coenzyme M-biosynthesis | 2.92 |
|  |  | pyridoxine 5-phosphate biosynthesis | 2.92 |
| Glycan biosynthesis | - | glycogen biosynthesis | 2.53 |
| Glycan metabolism | - | exopolysaccharide EPS-I-biosynthesis | 3.11 |
| Isoprenoid biosynthesis | - | geranylgeranyl diphosphate biosynthesis | 2.22 |
|  |  | isopentenyl diphosphate biosynthesis via mevalonate pathway | 2.09 |
| Lipid metabolism | - | mitochondrial fatty acid beta oxidation | 2.33 |
| Metabolic intermediate biosynthesis | - | 2-deoxystreptamine biosynthesis | 2.06 |
| Metabolic intermediate metabolism | - | (R)-mevalonate degradation | 2.95 |
|  |  | carbamoyl phosphate degradation | 2.73 |
| Nitrogen metabolism | - | urea cycle | 2.89 |
| Nucleoside biosynthesis | 2.21 | alpha ribazole biosynthesis | 2.21 |
| Nucleotide sugar biosynthesis | 4.30 | dTDP 4 acetamido 4 6-dideoxygalactose biosynthesis | 3.79 |
|  |  | dTDP 6-deoxy L-altrose biosynthesis | 3.79 |
|  |  | UDP alpha D-glucuronate biosynthesis | 3.51 |
|  |  | UDP L-arabinose biosynthesis | 3.04 |
| One carbon metabolism | - | methanol degradation | 2.50 |
| Phospholipid metabolism | - | decaprenyl phosphate biosynthesis | 2.23 |
| Porphyrin containing compound metabolism | - | bacteriochlorophyll biosynthesis | 2.28 |
| Protein biosynthesis | 2.43 | polypeptide chain elongation | 2.52 |
| Protein modification | - | NiFe hydrogenase maturation | 2.14 |
|  |  | protein lipoylation via exogenous pathway | 2.49 |
| Purine metabolism | 3.97 | AMP biosynthesis via de novo pathway | 3.37 |
|  |  | AMP biosynthesis via salvage pathway | 2.47 |
|  |  | GMP biosynthesis via salvage pathway | 2.52 |
|  |  | hypoxanthine degradation | 3.03 |
|  |  | IMP biosynthesis via salvage pathway | 2.82 |
|  |  | ppGpp biosynthesis | 2.59 |
|  |  | purine nucleoside salvage | 2.70 |
|  |  | xanthosine degradation | 2.43 |
| Pyrimidine metabolism | - | CTP biosynthesis via de novo pathway | 3.14 |
|  |  | UMP biosynthesis via de novo pathway | 3.52 |
|  |  | UMP biosynthesis via salvage pathway | 3.52 |
| Spore coat biogenesis | 2.56 | spore coat polysaccharide biosynthesis | 2.57 |
| tRNA modification | 3.00 | tRNA queuosine biosynthesis | 2.99 |
| Alcohol metabolism | - | ethanol biosynthesis via fermentation pathway | 2.09 |
| Amine and polyamine biosynthesis | - | ectoine biosynthesis | 2.63 |
| Antibiotic biosynthesis | - | streptomycin biosynthesis | 2.16 |
| Carbohydrate acid metabolism | - | 2-dehydro 3-deoxy D-gluconate degradation | 2.83 |
| Carbohydrate metabolism | - | galactose metabolism | 2.79 |
|  |  | glyoxylate and dicarboxylate metabolism | 2.84 |
|  |  | hexose metabolism | 2.44 |
|  |  | pentose and glucuronate interconversion | 3.16 |
| Cell surface structure biogenesis | 2.12 | S-layer biogenesis | 2.11 |
| Cofactor biosynthesis | - | 7-8-dihydroneopterin triphosphate biosynthesis | 2.68 |
|  |  | biotin biosynthesis | 2.51 |
| Cofactor metabolism | 2.47 | coenzyme M-coenzyme B heterodisulfide reduction | 2.55 |
| Fermentation | 3.96 | pyruvate fermentation | 3.95 |
| Glucan metabolism | 3.39 | xyloglucan degradation | 3.39 |
| Glycan biosynthesis | 2.75 | starch biosynthesis | 2.55 |
| Glycan degradation | - | starch degradation | 2.87 |
| Glycan metabolism | 3.45 | beta D-glucan degradation | 2.57 |
|  |  | cellulose degradation | 2.32 |
| Nucleotide sugar biosynthesis | - | CDP 3-6-dideoxy D-mannose biosynthesis | 3.47 |
|  |  | UDP alpha D-xylose biosynthesis | 2.63 |
| Photosynthesis | 3.19 | C4 acid pathway | 3.19 |
| Plant hormone biosynthesis | 2.17 | - | - |
| Plant hormone metabolism | 2.08 | - | - |
| Polyol metabolism | - | 1-2-propanediol degradation | 2.81 |
| Pyrimidine metabolism | 3.99 | dTMP biosynthesis via salvage pathway | 2.24 |
| Siderophore biosynthesis | - | rhizobactin biosynthesis | 2.13 |
| Steroid biosynthesis | 2.23 | - | - |
| Terpene metabolism | 2.35 | - | - |
| Xenobiotic degradation | - | toluene degradation (regulation) | 2.65 |
| Amine and polyamine biosynthesis | - | agmatine biosynthesis | 2.87 |
|  |  | putrescine biosynthesis via agmatine pathway | 2.89 |
| Amine and polyamine degradation | - | ethanolamine degradation | 2.44 |
| Amino acid biosynthesis | - | L-asparagine biosynthesis | 3.76 |
|  |  | L-lysine biosynthesis via AAA pathway | 3.02 |
|  |  | L-lysine biosynthesis via DAP pathway | 3.57 |
| Amino acid degradation | 4.11 | L-glutamate degradation via hydroxyglutarate pathway | 4.12 |
|  |  | L-threonine degradation via propanoate pathway | 3.97 |
| Antibiotic biosynthesis | - | phosphinothricin biosynthesis | 2.02 |
| Carbohydrate acid metabolism | - | L-idonate degradation | 2.25 |
| Carbohydrate biosynthesis | - | gluconeogenesis | 3.06 |
| Carbohydrate degradation | 4.32 | glycolysis | 3.63 |
|  |  | L-arabinose degradation via L-ribulose | 3.46 |
|  |  | L-fucose degradation | 3.92 |
|  |  | L-rhamnose degradation | 3.74 |
| Carbohydrate metabolism | - | L-rhamnose metabolism | 2.95 |
|  |  | mannose metabolism | 3.28 |
| Cofactor biosynthesis | - | iron sulfur cluster biosynthesis | 2.29 |
|  |  | pyridoxal 5-phosphate biosynthesis | 3.14 |
| Exopolysaccharide biosynthesis | 3.23 | colanic acid biosynthesis | 3.24 |
| Glycan degradation | 3.02 | xylan degradation | 2.17 |
| Glycan metabolism | - | L-arabinan degradation | 2.47 |
|  |  | pectin degradation | 3.29 |
| Nucleotide sugar biosynthesis | - | GDP L-fucose biosynthesis via de novo pathway | 3.42 |
| Protein modification | - | sulfatase oxidation | 2.96 |
| Quinol quinone metabolism | 3.35 | 1-4 dihydroxy 2-naphthoate biosynthesis | 3.29 |
| Siderophore biosynthesis | - | enterobactin biosynthesis | 2.07 |
| Sulfur metabolism | - | sulfite reduction | 2.35 |
| Alcohol metabolism | 2.54 | ethanol degradation | 2.37 |
| Amine and polyamine biosynthesis | 3.67 | 1-3-diaminopropane biosynthesis | 3.71 |
|  |  | betaine biosynthesis via choline pathway | 3.03 |
| Amine and polyamine degradation | 3.27 | putrescine degradation | 3.22 |
|  |  | stachydrine degradation | 2.04 |
| Amino acid biosynthesis | - | D-alanine biosynthesis | 2.23 |
|  |  | glycine biosynthesis | 3.18 |
|  |  | L-proline biosynthesis | 3.44 |
|  |  | L-threonine biosynthesis | 3.36 |
|  |  | L-tryptophan biosynthesis | 3.41 |
|  |  | L-tyrosine biosynthesis | 3.11 |
| Amino acid degradation | - | 4 aminobutanoate degradation | 2.59 |
|  |  | D-alanine degradation | 2.76 |
|  |  | L-alanine degradation via dehydrogenase pathway | 2.99 |
|  |  | L-arginine degradation via AST pathway | 2.83 |
|  |  | L-histidine degradation into L-glutamate | 3.38 |
|  |  | L-leucine degradation | 3.06 |
|  |  | L-lysine degradation via saccharopine pathway | 2.78 |
|  |  | L-phenylalanine degradation | 3.11 |
|  |  | L-proline degradation into L-glutamate | 3.31 |
|  |  | L-tryptophan degradation via kynurenine pathway | 2.32 |
|  |  | nopaline degradation | 2.19 |
|  |  | Other | 2.18 |
| Amino acid metabolism | - | tryptophan metabolism | 2.96 |
| Aminoacyl tRNA biosynthesis | 2.82 | selenocysteinyl tRNA(Sec) biosynthesis | 2.83 |
| Antibiotic biosynthesis | - | calcium dependent antibiotic biosynthesis | 2.43 |
|  |  | mitomycin C biosynthesis | 2.81 |
|  |  | penicillin biosynthesis | 2.01 |
| Aromatic compound metabolism | 4.38 | 3-4 dihydroxybenzoate biosynthesis | 2.49 |
|  |  | 3-phenylpropanoate degradation | 3.08 |
|  |  | 4 hydroxyphenylacetate degradation | 3.71 |
|  |  | anthranilate degradation via hydroxylation | 2.54 |
|  |  | benzene degradation | 2.72 |
|  |  | benzoate degradation via hydroxylation | 3.57 |
|  |  | beta ketoadipate pathway | 3.95 |
|  |  | naphthalene degradation | 2.25 |
|  |  | p cresol degradation | 2.60 |
|  |  | p cumate degradation | 3.18 |
|  |  | phenol degradation | 2.93 |
|  |  | phenylpropanoid biosynthesis | 2.09 |
|  |  | serotonin biosynthesis | 2.09 |
| Bacterial outer membrane biogenesis | - | LPS-lipid A biosynthesis | 2.63 |
| Carbohydrate metabolism | - | D-xylose degradation (regulation) | 2.20 |
|  |  | Entner Doudoroff pathway | 2.35 |
|  |  | glyoxylate cycle | 3.42 |
|  |  | tricarboxylic acid cycle | 3.75 |
| Carotenoid biosynthesis | 3.43 | staphyloxanthin biosynthesis | 3.44 |
| Cell wall biogenesis | - | peptidoglycan biosynthesis | 2.41 |
|  |  | peptidoglycan recycling | 2.02 |
| Cofactor biosynthesis |  | B6-vitamer interconversion | 2.70 |
|  |  | molybdopterin biosynthesis | 2.34 |
|  |  | NAD(+) biosynthesis | 3.47 |
|  |  | tetrahydrofolate biosynthesis | 3.08 |
|  |  | thiamine diphosphate biosynthesis | 3.03 |
|  |  | ubiquinone biosynthesis | 2.59 |
|  |  | ubiquinone biosynthesis (regulation) | 2.49 |
| Energy metabolism | - | oxidative phosphorylation | 2.58 |
| Flavonoid metabolism | 2.01 | quercetin degradation | 2.01 |
| Genetic information processing | 2.53 | DNA replication | 2.51 |
| Glycan biosynthesis | - | alginate biosynthesis (regulation) | 2.08 |
| Glycan metabolism |  | Vi antigen biosynthesis | 2.67 |
| Hydrocarbon metabolism | 2.79 | alkane degradation | 2.78 |
| Ketone degradation | 3.04 | acetoin degradation | 3.04 |
| Ketone metabolism | 2.75 | succinyl CoA degradation | 2.79 |
| Lipid metabolism | 3.49 | fatty acid beta oxidation | 3.29 |
|  |  | fatty acid metabolism | 2.24 |
|  |  | malonyl CoA biosynthesis | 2.58 |
|  |  | oleic acid biosynthesis | 2.07 |
|  |  | phospholipid metabolism | 2.26 |
|  |  | rhamnolipid biosynthesis | 2.25 |
|  |  | steroid degradation | 2.11 |
| Metabolic intermediate biosynthesis | 3.09 | (R)-mevalonate biosynthesis | 2.81 |
|  |  | chorismate biosynthesis | 2.82 |
|  |  | prephenate biosynthesis | 2.14 |
| Metabolic intermediate metabolism | - | (S)-3-hydroxy 3-methylglutaryl CoA degradation | 2.20 |
| Nitrogen metabolism | 3.91 | (S)-allantoin degradation | 3.82 |
|  |  | urea degradation | 3.37 |
| Nucleotide sugar biosynthesis | - | UDP N-acetyl alpha D-glucosamine biosynthesis | 3.04 |
| One carbon metabolism | 3.79 | formaldehyde assimilation via serine pathway | 3.03 |
| Opine metabolism | - | mannopine biosynthesis (regulation) | 2.38 |
| Organic acid metabolism | - | propanoate degradation | 2.78 |
| Organosulfur degradation | 2.67 | taurine degradation via aerobic pathway | 2.54 |
|  |  | Other | 2.12 |
| Phospholipid metabolism | 2.77 | CDP diacylglycerol biosynthesis | 2.54 |
|  |  | phosphatidylglycerol biosynthesis | 2.63 |
| Photosynthesis | - | C3-acid pathway | 2.03 |
| Pigment biosynthesis | 2.17 | betalain biosynthesis | 2.12 |
|  |  | ommochrome biosynthesis | 2.11 |
| Polyol metabolism | - | glycerol degradation | 2.54 |
| Porphyrin containing compound metabolism | 3.86 | chlorophyll biosynthesis | 2.80 |
|  |  | heme A biosynthesis | 2.54 |
|  |  | heme O biosynthesis | 2.39 |
|  |  | protoheme biosynthesis | 2.57 |
|  |  | protoporphyrin IX biosynthesis | 3.70 |
|  |  | siroheme biosynthesis | 2.73 |
| Protein modification | 3.20 | protein lipoylation via endogenous pathway | 3.29 |
| Purine metabolism | - | urate degradation | 2.74 |
|  |  | Other | 3.14 |
| Quinol quinone metabolism | - | menaquinone biosynthesis | 2.91 |
| Secondary metabolite metabolism | 2.71 | methylglyoxal degradation | 2.64 |
| Siderophore biosynthesis | 2.81 | bacillibactin biosynthesis | 2.03 |
|  |  | petrobactin biosynthesis | 2.61 |
|  |  | vibriobactin biosynthesis | 2.11 |
|  |  | vulnibactin biosynthesis | 2.12 |
| Sulfur metabolism | 2.61 | dibenzothiophene degradation | 2.37 |
|  |  | glutathione biosynthesis | 2.87 |
|  |  | hydrogen sulfide biosynthesis | 3.48 |
| Xenobiotic degradation | 3.82 | (2-4-5-trichlorophenoxy)acetate degradation | 2.08 |
|  |  | (2-4-dichlorophenoxy)acetate degradation | 2.51 |
|  |  | 4-nitrophenol degradation | 2.01 |
|  |  | atrazine degradation | 2.54 |
|  |  | benzoate degradation via CoA ligation | 3.16 |
|  |  | biphenyl degradation | 3.27 |
|  |  | carbazole degradation | 2.03 |
|  |  | dichloromethane degradation | 2.05 |
|  |  | pentachlorophenol degradation | 2.14 |
|  |  | polychlorinated biphenyl degradation | 3.17 |
|  |  | toluene degradation | 2.73 |
|  |  | vanillyl alcohol degradation | 2.19 |
|  |  | xylene degradation | 2.57 |
