# Supplementary material for: Gut microbiomes of wild great apes fluctuate seasonally in response to diet
Source: Nat Commun. 2018 May 3;9:1786. doi: 10.1038/s41467-018-04204-w (PMC5934369; doi:10.1038/s41467-018-04204-w)
Supplement: Supplementary file 10 — Supplementary Data 7 [file 41467_2018_4204_MOESM10_ESM.docx]

**Supplementary Data 7.** Unipathway superpathways and pathways (predicted by HUMAnN2) identified as associated with WLG Treponema-abundant enterotype 2 (red) or WLG Prevotella-abundant enterotype 3 (pink) samples by LEfSe analysis of the five WLG enterotype 2 and five WLG enterotype 3 samples selected for shotgun metagenomics sequencing.

| **Superpathway** | **LDA score** | **Pathway** | **LDA score** |
| --- | --- | --- | --- |
| Alkaloid biosynthesis | 2.79 | taxol biosynthesis | 2.27 |
| Alkene biosynthesis | 2.30 | ethylene biosynthesis via S-adenosyl L-methionine | 2.30 |
| Amine and polyamine biosynthesis | - | 1-3-diaminopropane biosynthesis | 2.73 |
|  |  | ectoine biosynthesis | 2.51 |
| Amino acid biosynthesis | - | L-pyrrolysine biosynthesis | 2.16 |
|  |  | L-threonine biosynthesis | 2.71 |
| Capsule biogenesis | 2.13 | capsule polysaccharide biosynthesis | 2.20 |
| Carbohydrate biosynthesis | - | Calvin cycle | 2.20 |
| Carbohydrate degradation | - | pentose phosphate pathway | 2.59 |
| Carbohydrate metabolism | - | galactose metabolism | 2.33 |
|  |  | glyoxylate and dicarboxylate metabolism | 2.49 |
| Carotenoid biosynthesis | 2.39 | - | - |
| Cell wall biogenesis | 2.81 | poly(glucopyranosyl N-acetylgalactosamine 1-phosphate) teichoic acid biosynthesis | 2.62 |
|  |  | poly(glycerol phosphate) teichoic acid biosynthesis | 2.13 |
| Cofactor biosynthesis | - | 7-8-dihydroneopterin triphosphate biosynthesis | 2.52 |
| Cofactor metabolism | 2.48 | coenzyme M coenzyme B heterodisulfide reduction | 2.48 |
| Glycan biosynthesis | 2.29 | starch biosynthesis | 2.29 |
| Glycan metabolism | - | beta D-glucan degradation | 2.38 |
|  |  | Vi antigen biosynthesis | 2.30 |
| Hydrocarbon metabolism | 2.13 | alkane degradation | 2.13 |
| Lipid metabolism | - | fatty acid metabolism | 2.14 |
| Nitrogen metabolism | - | urea cycle | 2.35 |
| Nucleotide sugar biosynthesis | - | UDP alpha D-xylose biosynthesis | 2.46 |
| One carbon metabolism | 3.54 | methanogenesis from methylamine | 2.92 |
|  |  | methanogenesis from methylated amine | 2.40 |
|  |  | methanogenesis from trimethylamine | 2.91 |
|  |  | methyl coenzyme M reduction | 2.85 |
| Photosynthesis | 2.78 | C4 acid pathway | 2.61 |
| Phytoalexin biosynthesis | 2.53 | 3-4-5-trihydroxystilbene biosynthesis | 2.13 |
| Plant hormone biosynthesis | 2.13 | - | - |
| Plant hormone metabolism | 2.08 | - | - |
| Pyrimidine metabolism | 3.72 | CTP biosynthesis via de novo pathway | 2.67 |
| Secondary metabolite biosynthesis | - | dhurrin biosynthesis | 2.18 |
| Siderophore biosynthesis | - | rhizobactin biosynthesis | 2.14 |
| Steroid biosynthesis | 2.04 | - | - |
| Terpene metabolism | 2.35 | - | - |
| Xenobiotic degradation | - | atrazine degradation | 2.19 |
| Amine and polyamine biosynthesis | - | agmatine biosynthesis | 2.70 |
| Amine and polyamine degradation | - | ethanolamine degradation | 2.38 |
| Amino acid biosynthesis | 3.79 | L-leucine biosynthesis | 2.57 |
|  |  | L-lysine biosynthesis via DAP pathway | 3.54 |
|  |  | L-tryptophan biosynthesis | 2.64 |
| Amino acid degradation | - | 4-aminobutanoate degradation | 2.03 |
|  |  | L-tryptophan degradation via pyruvate pathway | 2.39 |
| Amino sugar metabolism | - | N-acetylmuramate degradation | 2.16 |
| Aromatic compound metabolism | - | 3-4-dihydroxybenzoate biosynthesis | 2.17 |
| Carbohydrate acid metabolism | - | D-glucarate degradation | 2.67 |
| Carbohydrate biosynthesis | - | gluconeogenesis | 2.35 |
| Carbohydrate degradation | 3.74 | L-fucose degradation | 3.52 |
| Carbohydrate metabolism | - | L-rhamnose metabolism | 2.76 |
| Cofactor biosynthesis | - | pyridoxal-5-phosphate biosynthesis | 2.80 |
|  |  | thiamine diphosphate biosynthesis | 2.60 |
| Organic acid metabolism | - | 2-oxosuberate biosynthesis | 2.28 |
|  |  | glycolate degradation | 2.65 |
| Protein modification | 2.35 | sulfatase oxidation | 2.75 |
| Quinol quinone metabolism | 3.36 | 1-4 dihydroxy-2-naphthoate biosynthesis | 3.30 |
|  |  | menaquinone biosynthesis | 2.44 |
| Sulfur metabolism | - | hydrogen sulfide biosynthesis | 2.54 |
